# Behavioral differences between humans and machines arise early in visual processing

**DOI:** 10.1167/jov.26.2.9

**Published:** 2026-02-17

**Authors:** Thomas Klein, Wieland Brendel, Felix A. Wichmann

**Affiliations:** 1 Max-Planck-Institute for Intelligent Systems, Tübingen, Germany; 2 ELLIS Institute Tübingen, Tübingen, Germany; 3 Tübingen AI Center, Tübingen, Germany; 4 Department of Cognitive Science, University of Tübingen, Tübingen, Germany

**Keywords:** psychophysics, object recognition, DNN, error consistency

## Abstract

It remains an open question to what extent current deep neural networks (DNNs) are suitable computational models of the human visual system. While DNNs have proven to be capable of predicting neural activations in primate visual cortex with great success, psychophysical experiments have shown behavioral differences between DNNs and human observers. One of these behavioral differences is which individual images DNNs and human observers find easy or difficult to recognize, as quantified by *error consistency (EC)*. Hypothetically, the reported differences in EC could arise late in visual processing, even though the representations extracted by DNNs and human observers may have been more similar in the initial forward sweep: At the presentation and response times investigated in earlier work, observer-internal idiosyncrasies (e.g., in feedback-mediated memory) might have influenced the final behavioral responses, lowering EC between DNNs and human observers. To test this hypothesis, we systematically vary presentation times of backward-masked stimuli from 8.3 to 267 ms and measure human performance on a speeded eightfold identification task with natural images. Contrary to the hypothesis that error consistency peaks early in time, we find that it never exceeds the value of 0.4 known from previous work with longer presentation times, suggesting that the differences between DNNs and humans cannot be explained by late high-level reasoning but point to systematic processing differences between DNNs and the early human visual system.

## Introduction

How the brain recognizes objects from visual inputs is one of the central open questions of contemporary vision science, with a rich history of computational suggestions and ideas (Biederman, 1987; Hummel & Biederman, 1992; Bülthoff & Edelman, 1992; Peissig & Tarr, 2007; Gauthier & Tarr, 2016). One currently popular idea is that of the visual system as an initially (largely) feed-forward algorithm that extracts stable representations of the world that are invariant to identity-preserving transformations (Riesenhuber & Poggio, 1999), a highly nonlinear “untangling” transformation (DiCarlo & Cox, 2007; DiCarlo, Zoccolan, & Rust, 2012). It is hypothesized that such a nonlinear feed-forward transformation underlies rapid object recognition: One of the striking empirical findings regarding human object recognition is that under typical viewing conditions, object recognition is very fast (Biederman, 1987; Thorpe, Fize, & Marlot, 1996). In binary tasks such as animal detection, it has been shown that the presence or absence of the target can be decoded from electroencephalogram (EEG) signals about 150 ms after stimulus onset, for images that were shown for only 20 ms (Thorpe et al., 1996). These EEG signals not only reflect low-level statistical differences between different types of stimuli but also are truly correlated with behavior, with a low-level perceptual mechanism completing even faster, at about 80 ms after stimulus onset (VanRullen & Thorpe, 2001).

Building artificial systems capable of recognizing objects with human-like accuracy used to be an elusive goal, until deep neural networks (DNNs) revolutionized the field of computer vision. DNNs not only enable computers to classify objects in natural images (Krizhevsky, Sutskever, & Hinton, 2012; He, Zhang, Ren, & Sun, 2016) but also learn generally useful representations of visual inputs (Rumelhart, Hinton, & Williams, 1986; Sharif Razavian, Azizpour, Sullivan, & Carlsson, 2014; Donahue et al., 2014). Having access to image-computable models that achieve human-like performance on classification tasks inspired the research program of neuroconnectionism: using DNNs as a class of computational models that can express falsifiable hypotheses of the ventral stream (Doerig et al., 2023). To us, two positive applications of DNNs in vision science are uncontroversial: First, they are powerful tools to study vision with a multitude of successful applications (Wichmann & Geirhos, 2023). Second, they have great potential as explorative models (Cichy & Kaiser, 2019; Wichmann & Geirhos, 2023). What remains an open question, however, is whether current DNNs are already adequate models of visual object recognition (Bowers et al., 2023; Wichmann & Geirhos, 2023). The answer to this question notwithstanding, the discussion should center on *how* DNNs should best be used in vision science and not *whether* they should be used (Funke et al., 2021; Firestone, 2020; Lonnqvist, Bornet, Doerig, & Herzog, 2021; Bowers et al., 2023; Wichmann & Geirhos, 2023).

Perhaps the strongest argument in favor of DNNs as computational models of human object recognition is their predictive power over neuronal signals: The neural activation elicited by a specific image in a primate brain can be predicted with great accuracy from the activation that the same image elicits at a specific DNN layer (Yamins et al., 2014; Kriegeskorte, 2015; Kubilius et al., 2019; Zhuang et al., 2021). One can either directly predict neural activations from DNN activations for the same stimuli (Kubilius et al., 2019; Allen et al., 2022; Willeke et al., 2023) or analyze the similarity between the representations extracted by the two systems, for example, using (variants of) representational similarity analysis (RSA) (Kriegeskorte, Mur, & Bandettini, 2008; Khaligh-Razavi, Henriksson, Kay, & Kriegeskorte, 2017; but see also Dujmović, Bowers, Adolfi, & Malhotra, 2023). Progress on DNN predictions of neural data is monitored by the popular Brain-Score benchmark (Schrimpf et al., 2018), and although it is not quite clear which specific model should be considered most brain-like overall, because performance on the different measures of alignment is not highly correlated (Ahlert, Klein, Wichmann, & Geirhos, 2024), Brain-Score is completely dominated by DNN models of vision, instead of hand-crafted mechanistic models.

On the other hand, there is a growing body of work finding behavioral differences between modern DNNs and human observers. While not all differences in visual processing are necessarily relevant (Lonnqvist et al., 2021), and one has to be careful not to confuse differences in performance with differences in competence (Firestone, 2020), there are glaring discrepancies between DNNs and humans, such as DNNs’ susceptibility to adversarial perturbations (Goodfellow, Shlens, & Szegedy, 2015) that are imperceptible to humans. DNNs have been found to exhibit a texture bias that humans do not share (Geirhos et al., 2019), and they do not exhibit the same kind of configural sensitivity as human observers (Baker & Elder, 2022). Ollikka, Abbas, Perin, Kilpeläinen, and Deny (2024) recently showed that recognizing object categories from unusual viewpoints is difficult for DNNs (Abbas & Deny, 2023; Dong et al., 2022), which is typically easy for humans given sufficiently long viewing time. Similarly, DNNs were inferior to human observers at evaluating multiview object consistency (Bonnen et al., 2024) once humans were exposed to images for longer than 100 ms. DNNs also appear insensitive to realistic object scale, finding giant objects in scenes where humans tend to miss them (Eckstein, Koehler, Welbourne, & Akbas, 2017). DNNs are also less robust to image corruptions than human observers and have exhibited error patterns that differ from those of humans (Dodge & Karam, 2017; Geirhos et al., 2018). Furthermore, the susceptibility to different types of noise differs between DNNs and humans, with DNNs being more susceptible to spatially uncorrelated white noise, while humans are more susceptible to spatially correlated noise (Jang, McCormack, & Tong, 2021). In addition to rather subtle behavioral differences (Neri, 2022), the representations learned by humans also seem to differ from those learned by DNNs (Muttenthaler, Dippel, Linhardt, Vandermeulen, & Kornblith, 2023), and DNN representations also differ among themselves (Mehrer, Spoerer, Kriegeskorte, & Kietzmann, 2020). These differences seem to be modulated by training objective and dataset choice rather than model architecture and scale (Muttenthaler et al., 2023), and better-performing models are not necessarily more aligned to human observers than worse-performing ones (Fel, Rodriguez, Linsley, & Serre, 2022). Notably, there have also been successful attempts to deliberately align DNNs with human observers, beginning with Peterson et al. (2018). The proposed methods for increasing alignment range from combinations of different training tricks (Riedel, 2022) to directly integrating a model's ability to predict neural data into its objective function (Dapello et al., 2023).

Earlier work investigating the behavioral similarity between DNNs and human observers (Rajalingham et al., 2018; Geirhos et al., 2021; Ollikka et al., 2024) found striking *error inconsistencies*, suggesting that different strategies are employed for the task of object recognition by humans and machines. Error consistency between two observers is measured by presenting both observers with a sequence of natural images, each of which belongs to one of *N* nonoverlapping categories (i.e., images have exactly one ground-truth label). Observers are tasked with correctly classifying the images, analogous to how DNNs are typically evaluated on benchmark datasets such as ImageNet-1k (Russakovsky et al., 2015). Next, the correctness of each individual classification response is recorded to evaluate whether the two observers make mistakes on the same images. Error consistency is then quantified by a scalar value, denoted *κ*, which we explain in more detail in Quantifying error consistency. The idea behind error consistency is that of “molecular psychophysics” (Green, 1964): When vision models were still performing much worse on standard benchmark tasks than human observers, one could reasonably consider the best-performing model to be the “most human-like” model. But now that models perform on par with humans, inviting hypotheses about their internal processing being similar to that of humans, we have to find a measure of similarity that allows a more fine-grained analysis, down to the level of individual trials.

Geirhos et al. (2021) found that human observers were consistent in their mistakes (i.e., they systematically misclassified the same images). Analogously, the investigated DNNs were found to be largely consistent among themselves as well. But between the two groups, a large behavioral gap was found: Humans and machines make errors on different images, hinting at differences between the two types of systems.

The apparent discrepancy between the *strong predictive power of DNNs*, on the one hand, and the *error inconsistencies between humans and DNNs*, on the other, needs to be resolved to clarify the role that DNNs can play in understanding human object recognition: Are they merely a *tool* to study object recognition, or could they be a *model* of human object recognition? As Bowers et al. (2023) and Guest and Martin (2023) point out, predictive power alone is insufficient to suggest strong functional similarity between two systems. For example, a digital watch can be a great predictor of an analogue watch without sharing any of its operating principles (Guest & Martin, 2023). On a similar vein, chess computers achieve performance equaling or surpassing that of the best human players by employing robotic playing styles that feel distinctly unnatural and are measurably different from human style (Regan, Biswas, & Zhou, 2014). Simply put, there may be different means of achieving the same computational ends. This objection is important to keep in mind, because even though DNN classification performance keeps increasing to the point of saturating standard benchmarks (Beyer, Hénaff, Kolesnikov, Zhai, & van den Oord, 2020), DNN architectures have changed dramatically in recent years: While early DNNs like AlexNet (Krizhevsky et al., 2012) were explicitly modeled after the visual system, modern architectures like Vision Transformers (ViTs) (Dosovitskiy et al., 2021) no longer share this structural similarity with the brain. We would expect a good model of a system to not only be predictive of intermediate, internal states of the system but also to be able to reproduce characteristic aspects of its behavior, such as which natural images of objects it finds easy or difficult to recognize. Exactly which aspects of the human visual system are characteristic (Lonnqvist et al., 2021) and which level of analysis is sufficient to conclude that characteristics are reproduced (Neri, 2022) is an important ongoing debate. We argue that agreeing upon whether an image of an object is easy or difficult to recognize is surely an important characteristic aspect that a good model of human object recognition should be able to reproduce.

**Figure 1.**
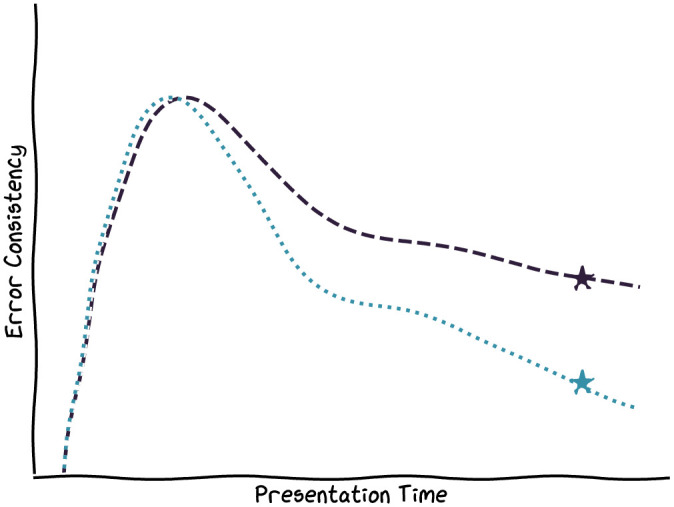
**Sketch of how results might look if the “reconciliation hypothesis” were true.** We sketch human–human EC in dark blue and human–machine EC in light blue. Stars indicate results from earlier work, recorded at a 200-ms presentation time (perhaps already too long in the context of object recognition) and with a slower mouse-click response modality. At extremely short presentation times, when humans do not perceive the stimuli and answer randomly (and thus independently from one another), error consistency should be close to zero. At some intermediate presentation time, when a forward pass of the DNN models the forward pass through the ventral stream well, error consistencies should peak. Finally, at longer presentation times, when observer-internal idiosyncrasies (arising, for example, from feedback-mediated memory) influence the final behavioral responses, humans and machines might diverge, leading to an underestimation of human–DNN consistency in visual processing.

One hypothesis that could reconcile this discrepancy is that while DNNs might indeed be good models of initial visual processing, higher-level, possibly recurrent (Kietzmann et al., 2019) cortical processing of the extracted representations could ultimately cause the observed behavioral differences—the “reconciliation hypothesis.” To investigate this hypothesis, we here conducted further behavioral experiments in which we drastically reduced the presentation and response times of human observers and investigated the error consistencies between humans and a suite of 10 different DNNs as a function of presentation time. To suppress further processing of the stimuli by human observers, we employed a high-contrast backward mask and collected speeded responses with a research-grade touchscreen, reducing the time required to give responses by 20% relative to earlier work (694 ms median response time [RT] instead of 863 ms). If it were true that higher-level cortical processing was the root cause of human–machine error inconsistencies, we would expect time-constrained human observers to behave more like machines and error consistencies to increase as stimuli are presented for progressively shorter amounts of time, at least until human observers are no longer able to recognize the stimuli. We visualize the results expected under this hypothesis in Figure 1.

## Methods

To investigate the relationship between stimulus presentation time and human–machine error consistency, we conducted an experiment inspired by Geirhos et al. (2021) but differing with respect to multiple crucial aspects: We presented human observers with (corrupted) natural images drawn from the training set of ImageNet-1k, which we grouped into super-classes at the *basic level* of categorization (Rosch, Mervis, Gray, Johnson, & Boyes-Braem, 1976; Jolicoeur, Gluck, & Kosslyn, 1984) that is most easily accessible to humans. We then asked human observers to classify these images into one of eight categories, giving us data about which images humans make mistakes on. Unlike earlier studies, our main goal was to aggressively speed up the responses to suppress higher-level processing as much as possible. To this end, we employed a research-grade touchscreen allowing for much faster response times than mouse clicks, limited the time available for giving responses to only 800 ms, and reduced the number of categories from 16 down to 8 to render the task easier. These experimental design choices proved successful, reducing the median response time to 694 ms (169 ms faster than the median RT of earlier work). Conducting a median split of the data reduced RTs even further, by 80 ms on average, without meaningfully changing our results. We conducted experiments at six different presentation times, ranging from 8.3 to 266.67 ms. For reference, Geirhos and colleagues used a fixed presentation time of 200 ms, corresponding to a lower bound of the typical length of one fixation (DiCarlo et al., 2012). We then calculated error consistency (EC) between DNNs and humans at each presentation time, as described in Quantifying error consistency. All code, data, and other resources necessary to reproduce our experiments are available at https://github.com/wichmann-lab/early_behavioral_differences.

### Stimuli

In principle, error consistency could be calculated on any set of labeled images, but there are some practical limitations, such as the maximum number of different categories that human observers can reasonably be asked to discern. Evidence from psychological literature (Rosch et al., 1976; Jolicoeur et al., 1984) shows that humans give the fastest responses to objects at the entry level of taxonomies. For example, a human asked to classify a songbird would typically think of the basic level (“bird”) first, rather than sub- or superordinate levels (e.g., “robin” or “animal”). Hence, to optimize response speed, we grouped the subordinate ImageNet categories into eight classes at the basic level, closely following earlier work (Geirhos et al., 2018; Geirhos et al., 2021) that used 16 groups, which we deemed too many for the kinds of high-speed responses we required. To create our dataset of stimuli, we first took a closer look at the data from Geirhos et al. (2018) and found that 9% of the classification mistakes on uncorrupted images turn out to be artifacts caused by the suboptimal labels of the ImageNet dataset: Of the 3, 259 images shown without corruptions in earlier experiments, only 788 were misclassified at least once by any of the human observers, and at least 74 of those should be dismissed, since they contain some form of label ambiguity—one of many known problems with ImageNet labels (Beyer et al., 2020). We exemplify prototypes of these problems in Supplementary Figure S1. To address this issue, we obtained an updated set of stimuli via a three-stage process. First, we reduced the original set of 16 categories to a set of 8 unambiguous categories (airplane, boat, car, bike, elephant, bear, dog, bird; see Supplementary Appendix A for details). Second, we manually removed all images containing any ambiguity, label errors, or other issues, resulting in about 900 candidate images for each class. This was done by visually inspecting about 8,000 candidate images and keeping only those that do not violate the criteria listed in Supplementary Appendix A.

If these images were presented to the DNNs without corruptions, the DNNs would reach very high accuracies (between 98.2% and 100%), because they were trained on ImageNet-1k itself or on even larger datasets of natural images. The categories in ImageNet are not equally difficult (Cui et al., 2024), and presumably, our eight categories are relatively easy, especially after the removal of ambiguous images. However, the calculation of error consistency requires that sufficiently many mistakes are made on the dataset, since for even just one observer near ceiling performance, a single changed answer can have a substantial impact on EC, meaning that error consistency—like many statistics—will not work reliably near floor or ceiling performance. In other words, we need to induce classification errors in the models, ideally without affecting humans too much. As Dodge and Karam (2017) have found, additive noise and blur have the effect of decaying model performance more than human performance, as also confirmed by Geirhos et al. (2018). Training models on these corruptions has been shown to recover performance and yield models that can be used to predict image-level performance thresholds of humans (Jang et al., 2021; Jang & Tong, 2024).

**Figure 2.**
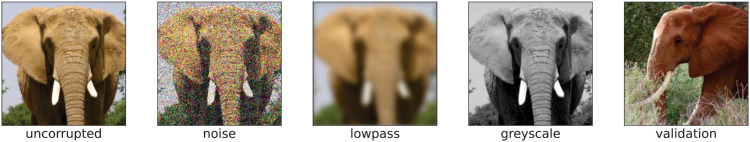
**Illustration of image corruptions.** The strengths of the parameterized corruptions (noise and lowpass) were chosen to deteriorate model performance as much as possible without reducing human performance more than necessary.

We therefore corrupted the images by either applying a lowpass filter or adding uniform noise, resulting in much lower accuracies for most models (see Figure 2 for an illustration and Figure 5 for an overview of model performance on the corrupted images). A potential drawback of this approach is that it remains unclear to what extent human–machine differences found in this domain will transfer to other domains, such as uncorrupted images, or different corruptions. An alternative approach would have been to present uncorrupted images but inject noise into the activations at deeper layers of the DNN. However, it is not clear that this would truly constitute a better comparison, as it arguably changes the model. We argue that even if behavioral differences between humans and machines only materialize in some domains, they would be indicative of computational differences, and we can investigate at which stage of processing these differences arise.

To corrupt images with noise, uniform noise sampled from the interval [−0.5, 0.5] was added to all three channels of the image, before all values were again clipped to the interval [0, 1]. The lowpass filtering was implemented by convolving the images with a Gaussian kernel, *σ* = 5.0, where the image was padded with gray pixels. Again, as a last step, all image values were clipped to [0, 1]. The parameters of the corruptions (width of the uniform noise and *σ* of the Gaussian) were chosen via grid search as the minimum values reducing the classification performance of the most modern group of DNNs we investigated to below 90% (86.63% on noisy and 82.6% on lowpass-filtered images; see Supplementary Figure S3). We also measured performance on grayscale images to investigate the role color plays for image classification and also evaluated the models on images drawn from the validation set of ImageNet-1k (see Images should be drawn from the validation set).

**Figure 3.**
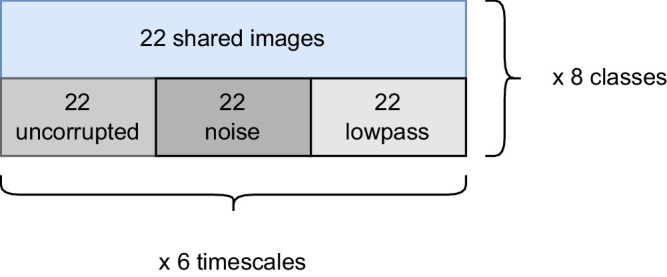
**Illustration of image allocation. For each category, we selected 88 images to be shown per presentation time.** Of these, 22 were shown with all three corruptions, while the others were only shown with one corruption applied. For the shortest and longest presentation times, when observers were near chance and ceiling performance, we selected only half as many images (i.e., 44 in total per class) to keep the experiment shorter.

For our human experiments, we first allocated 88 images of each category to each of the six time scales. Of these, 22 images were shared across corruptions, while for each corruption, 22 nonshared images were selected (see Figure 3). For every observer, the images of a time scale and corruption were shuffled randomly. A few randomly selected images were shown repeatedly to each observer, allowing us to gauge their self-consistency.

### Stimulus presentation

**Figure 4.**
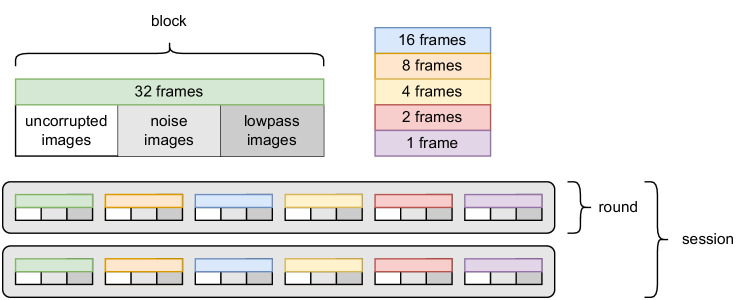
**Overview of the experiment.** Observers took part in two sessions on two different days, with each session consisting of two rounds of six blocks each, one for each presentation time.

Earlier work by Geirhos and colleagues presented stimuli on a ViewPixx monitor and recorded responses via mouse clicks. However, a crucial aspect of our experiments is the speed at which responses are given, which prompted us to employ a research-grade touchscreen, which allows for faster responses (rendering our median response time 169 ms faster). All experiments were conducted in a dark cabin, using a 22-inch VIEWPixx 3D light LCD monitor (VPixx Technologies, Saint-Bruno, Canada) at a refresh rate of 120 Hz (scanning backlight mode on). Responses were recorded with the TOUCHPixx accessory.^1^ The screen measured 484 × 302 mm, at a resolution of 1,920 × 1,200 pixels. Stimuli were presented foveally in the center of the screen. Ideally, in line with earlier work and common practices, we would show the stimuli in foveal vision, at no more than 3° of visual angle. However, due to the close proximity to the screen necessitated by the input modality and the relatively low pixel density of the monitor, doing so would force us to downscale images by a factor of 6. If we instead opt for 6° of visual angle, still well within paracentral vision and only slightly larger than what is usually considered central vision, we arrive at a stimulus size of 45 mm in diameter. This corresponds to a 180 × 180 pixel area, implying a scaling factor of only 1.5 for our stimuli of size 224 × 224, which we deemed acceptable. The background was set to a gray value of 0.454 in the [0, 1] range, in line with earlier experiments. A chin rest was used to maintain a fixed viewing distance and angle. The experiment was implemented using the Psychophysics Toolbox (Kleiner et al., 2007) (Version 3.0.12) in MATLAB (Release 2016a; The MathWorks, Natick, MA, USA) using a 12-core desktop computer (AMD HD7970 graphics card “Tahiti” by AMD, Sunnyvale, CA, USA) running Kubuntu 14.04 LTS. After an accommodation and training phase of 15 to 20 minutes, in which observers practiced using the touchscreen and memorized the button positions, the proper experimental trials began. Trials were split into two *sessions*, each of which took about 2 hours, so observers visited the lab twice. See Figure 4 for an illustration. Each session was again split into two *rounds*, in which observers saw stimuli at all presentation times and in all corruptions. In each round, there were six *blocks* of trials at a fixed presentation time. We started with the longest time scale, which should be the easiest condition. In each block, we showed images in three *sets* of corruptions, always in the same order: We started with uncorrupted images, followed by noisy images and finally lowpass-filtered images, so that observers had to readapt to new presentation times and corruptions as seldom as possible and knew what to expect (Blackwell, 1952). Additionally, the first three uncorrupted images of each block were practice images for which responses were discarded, to avoid artifacts caused by the abrupt change in presentation time. All sets in a block contained the same number of images, but we presented only 48 images per set in the first and last blocks and 96 images per set in the other blocks. In total, every observer completed 2 * 2 * (2 * 3 * 48 + 4 * 3 * 96) = 5,760 trials. While the sets themselves simply ran through without an option to stop, observers needed to start the next set manually, giving them a chance to take a break. We encouraged observers to take at least a minute-long break between sets to close and rest their eyes and to accommodate elsewhere.

Every trial consisted of a 250-ms fixation square, followed by the stimulus image (shown for the respective number of frames), and a 200-ms grayscale pink noise mask.^2^ Immediately after the mask, the response buttons appeared (slightly offset to the left or right, depending on the observer's handedness, for better ergonomics). Responses had to be given within 800 ms after stimulus offset. For the two longest presentation times, we repeated the experiment with 1,500 ms to reduce the proportion of random errors (see Supplementary Appendix B for details). Participants tapped a symbol on the touchscreen to indicate their response, which then lit up to confirm the contact. No correctness feedback was given to avoid response bias caused by feedback-induced changes in behavior and to minimize differences between human and DNN performance assessment. Participants could theoretically change their response arbitrarily many times, which in practice might be once or twice (we did not record this but estimate that it happened on ≤5% of trials). Reaction times were measured from the instant the buttons appeared until the first press of the final response (i.e., if observers double-tapped the same response, we recorded the first contact, but if they switched their answer, we recorded the first contact of the new answer).

### Deep neural networks

**Figure 5.**
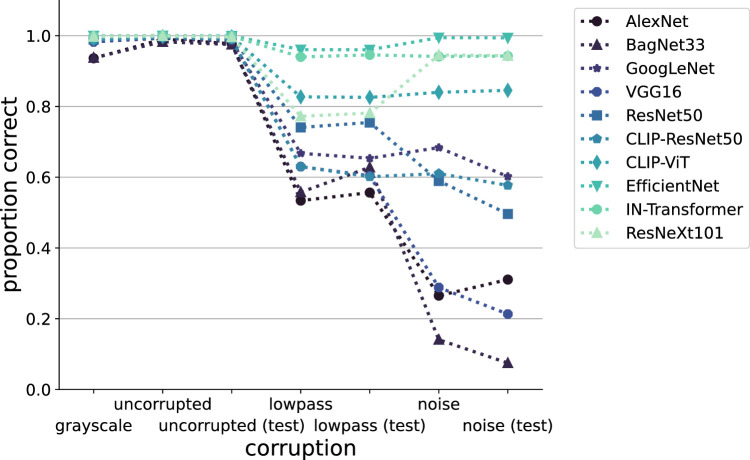
**Model performance on corrupted images.** For all 10 models, we plot their average accuracy for the different corruptions we investigate. Evidently, the four classic CNNs are far less robust than the five modern models, apart from the CLIP-trained ResNet50. Especially for the EfficientNet and the ImageNet-21k–trained ViT, performance hardly decays.

We compare our human observers against a suite of 10 DNNs taken from the *model-versus-human* benchmark (Geirhos et al., 2021), which differ with respect to various network design choices, resulting in differing performance in terms of classification accuracy under corruptions (see Figure 5).

Four of the investigated models are convolutional neural networks (CNNs) that were trained on ImageNet-1k in a supervised fashion (i.e., they were trained to predict category probabilities based on ground-truth labels). They comprise AlexNet (Krizhevsky et al., 2012), VGG (Simonyan & Zisserman, 2015), GoogLeNet (Szegedy et al., 2015), and ResNet-50 (He et al., 2016). While no longer state-of-the-art, these models are “classics” that are well known in the vision research community and investigated in related works (Peterson et al. 2018). The convolutional architecture shared by these models is biologically inspired, with convolutional filter kernels resembling local receptive fields in the visual cortex (LeCun, Bottou, Bengio, & Haffner, 1998). The information processing in these networks is mostly serial (i.e., data are processed in a purely “feed-forward” manner, apart from some parallel streams in the GoogLeNet and skip connections in the ResNet) and modulated by nonlinearities after every layer. These four CNNs span 4 years of developmental progress but mostly with respect to model architecture, not training paradigm.

In recent years, the biggest performance gains of DNNs on standard benchmarks have been achieved by drastically increasing the scale of models and training datasets (Kaplan et al., 2020), as well as by employing modern training paradigms. To investigate the effects that these developments have had on error consistency, we also consider a set of five more modern DNNs, which achieve or even surpass human-level performance on ImageNet. This set contains another ResNet-50 (whose architecture exactly matches the first one) that was trained using the CLIP objective (Radford et al., 2021). CLIP is a training procedure that requires a dataset of captioned images (i.e., every image in the training set is accompanied by a *caption*, a text snippet describing the image). A CLIP-trained model consists of two encoders: one encoder for the image (a model mapping an image to an embedding vector, e.g., a CNN) and another encoder for the caption, typically a Transformer (Vaswani et al., 2017). During training, the model is taught to embed images and captions into a common space so that image embeddings are close to the embeddings of their corresponding captions. The proprietary dataset used to train our CLIP models consists of 400 million image–text pairs collected from the Internet (i.e., two orders of magnitude larger than ImageNet-1k, on which the four classic CNNs were trained).

Another modern training paradigm is semi-supervised learning, an approach that combines large, unlabeled datasets with typically smaller but higher-quality labeled datasets. Yalniz, Jégou, Chen, Paluri, and Mahajan (2019) refine this approach to semi-weakly supervised learning (SWSL) by first pretraining a teacher-model on a large (940 million samples) dataset with low-quality labels before fine-tuning the teacher on ImageNet. Next, they use this teacher to create better labels for the large dataset and train a student-model on this improved training set. We investigate a ResNeXt (Xie, Girshick, Dollár, Tu, & He, 2017), a modernized variant of residual networks, that was trained using this paradigm.

We also investigate two Vision Transformers (Dosovitskiy et al., 2021), one of which was trained in a supervised fashion on ImageNet-21k (Ridnik, Ben-Baruch, Noy, & Zelnik-Manor, 2021) (the larger dataset of which ImageNet-1k is a subset) while the other one was trained using CLIP. Vision Transformers build on the Transformer architecture (Vaswani et al., 2017), which was originally proposed for highly parallel sequence processing, seeing application especially in the context of large language models (LLMs) (Devlin, Chang, Lee, & Toutanova, 2019; Radford et al., 2019) underlying modern chatbots like Chat-GPT. ViTs transfer this idea to the visual domain by dividing every input image into smaller patches and processing them in parallel using attention mechanisms. We also test an EfficientNet (Tan & Le, 2019) trained as a noisy student (Xie, Luong, Hovy, & Le, 2020), which was found by Abbas and Deny (2023) to be relatively robust to viewpoint changes. The EfficientNet architecture was, as the name suggests, designed to utilize the available computational power as efficiently as possible, incorporating components originally designed for resource-constrained environments and finding optimal ratios between network depth and layer width, for example. Finally, we consider BagNet (Brendel & Bethge, 2019) as a form of sanity check, a model that is deliberately prevented from using global features of the data and should theoretically employ strategies very different from human observers.

Except for the CLIP-trained networks, all of these models already support ImageNet-1k classification (i.e., they output a 1,000-dimensional vector of category probabilities). For the CLIP-trained models, we follow the standard evaluation paradigm of transforming the cosine similarities between image embeddings and text embeddings of the category (“An image of an X”) into category probabilities via softmax activation. We then calculate the probability of each of our eight super-categories as the average probability of the constituent ImageNet categories, following Geirhos et al. (2018), who proved that this aggregation strategy is the optimal way of dealing with shifted priors over classes at test time. In Figure 5, we plot the eight-way classification performance of our suite of models under the different image corruption types. The very broad range of accuracy values under corruptions (i.e., the differences in robustness of the different models) highlights the inherent diversity of DNN models, providing a cautionary tale about overly general statements about DNNs: They form a rather heterogeneous group of models.

### Quantifying error consistency

To quantify the behavioral consistency between two observers, we follow Geirhos, Meding, and Wichmann (2020), as well as Klein, Meyen, Brendel, Wichmann, and Meding (2025), and calculate error consistency using Cohen's kappa (Cohen, 1960). Cohen's kappa quantifies the agreement between two observers as

(1) $$\begin{array}{c} \kappa =\frac{p_{o}-p_{e}}{1-p_{e}} \end{array}$$

where *p_o_* is the proportion of trials for which both observers gave the same response (the *observed agreement*), while *p_e_* is the *expected agreement*: the proportion of trials for which equal responses are expected by chance alone. To calculate the chance agreement, observers are assumed to be independent binomial observers that treat every trial as a Bernoulli trial, where the *p* of their binomial distributions is given by their marginal response probabilities. Intuitively, *κ* quantifies how much more—or less—agreement is observed between the two observers than would have been expected by chance alone, relative to how much more agreement there could have been. For observers with equal marginal response distributions, *κ* takes on values in [−1, 1], with a value of 1.0 indicating perfect agreement between them, a value of 0.0 indicating chance agreement, and negative values indicating systematic differences. For observers with different marginal response distributions, however, the bounds for *κ* can change quite drastically—see Klein et al. (2025) for an in-depth discussion. To calculate *κ*, we ask both observers to classify all images. We are interested in which images each observer found “easy” or “difficult,” so we compare the given responses to the ground-truth labels, resulting in a binary sequence of correct/incorrect judgments by both observers, over which we then calculate *κ*. Thus, error consistency measures the observers’ agreement in the sense that it quantifies the proportion of images that were either easy for both (both gave a correct response) or difficult for both (both gave an incorrect response). An important corollary of EC's dependence on the marginals of the two observers is that error consistency is not orthogonal to accuracy (Klein et al., 2025): Two observers who are similarly accurate have a higher (theoretical) maximum error consistency than two observers who differ in their accuracy. If one wanted to measure the behavioral similarity of a pair of observers *irrespective of their accuracy*, error consistency would not be the right tool for the job—one would at least have to attempt some form of correction. However, it is not obvious that this orthogonality to accuracy is a necessary or even desirable property of a measure of behavioral similarity, since two observers who achieve very different accuracies are indeed less similar than two roughly equally correct observers. A necessary requirement for the sensible application of error consistency is not only that the very same stimuli are shown to both observers but also that the presented stimuli are heterogeneous in their difficulty: There must be easy and difficult stimuli. Were the observers’ performance solely determined by their internal noise, as is typical in low-level detection or discrimination tasks at a constant stimulus difficulty level, we would not expect observers to be consistent in their responses to individual stimuli (Green, 1964). If the task were too easy and observers made too few mistakes, EC would become an unstable measure requiring many trials and many observers to obtain stable estimates of the average, because even a single changed decision can drastically change the final *κ*. For example, if in a task with 100 images, both observers got 49 images right and 49 wrong, with diverging mistakes on only two images, adding one more image on which both observers fail would only very slightly increase their EC, from 0.96 to 0.9604. But if they both got 96 images correct and one wrong, again with two diverging mistakes, adding one more image on which both fail would drastically increase their EC, from 0.49 to 0.66. In practice, this issue is only noticeable at extremely low or high error rates. Earlier work used stimuli sets containing ambiguous, error-inducing images and therefore failed to notice this problem. Our procedure of manually cleaning the stimuli revealed this issue, necessitating the application of corruptions to reduce performance sufficiently to calculate stable error consistency values.

For a more detailed discussion of idiosyncrasies of error consistency and a survey of alternatives, we refer the interested reader to Supplementary Appendix C.

## Results

### Rapid object recognition in humans

**Figure 6.**
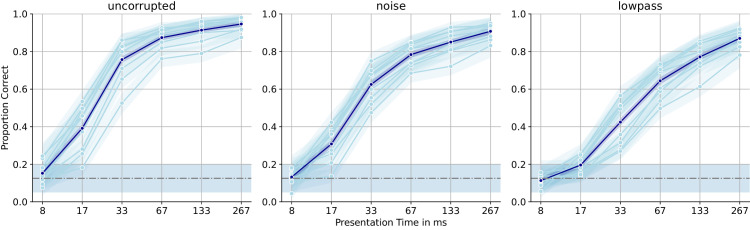
**Performance comparison of all observers.** We plot presentation time against classification performance. Every observer's average classification performance is a light blue line, with shaded regions indicating the 95% confidence interval. The average over all observers is given as the dark blue line. Across all corruptions, human observers benefit from longer presentation times. There are pronounced differences between observers, likely because of differences in alertness or motivation. Apparently, the *σ* chosen for the lowpass filtering resulted in stimuli that are comparatively harder for human observers.

**Figure 7.**
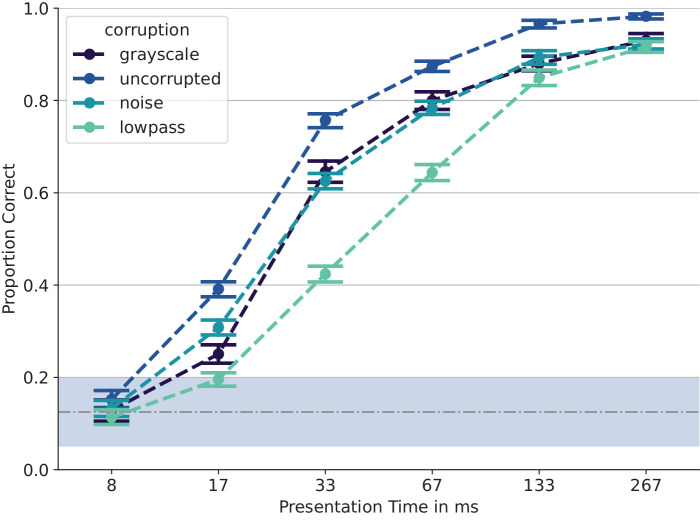
**Performance breakdown by corruption.** We plot presentation time against classification performance for the different corruption types. At maximum presentation time, the corrupted images are similarly difficult, but lowpass-filtered images seem to require much longer processing times for humans to process them successfully.

As expected, human observers benefit from longer presentation times but already reach 40% accuracy for a stimulus duration of only 17 ms (see Figure 6)—chance performance would be 12.5% for the eightfold identification task. At 32 frames (267 ms), performance on uncorrupted images is essentially at ceiling (94.65% correct), while for noisy and lowpass-filtered images, even longer presentation times might have improved performance further (90.76% and 87.01% correct, respectively). We plot aggregated performance across presentation times for all image corruptions in Figure 7 to show that the different corruptions affect the required processing time somewhat differently. Uncorrupted images are easiest, as expected, and all corruptions are similarly hard if observers have enough time, showing that our approach to balancing the corruption intensities (see Stimuli) successfully yielded similarly difficult conditions. But at shorter presentation times, lowpass-filtered images are noticeably harder than noisy and grayscale images, inviting speculation about more top-down processing being required to make sense of such images: Arguably, noise could be removed via bottom-up mechanisms, as has been suggested previously (Atick & Redlich, 1992; Martinez-Garcia, Cyriac, Batard, Bertalmío, & Malo, 2018). But to “reintroduce” some of the information about the high-frequency details that were removed through low-pass filtering, some top-down method might be required. Notably, our eight observers exhibit fairly pronounced interpersonal performance differences: In Supplementary Figure S7, we show a breakdown of human performance by image corruption, revealing that human performance is consistent across corruptions (i.e., observers who perform well on the uncorrupted images tend to also perform well on the harder lowpass-filtered images).

In line with other experiments, our observers are able to solve the task very quickly: We measure a median RT of 694 ms (169 ms faster than the median RT in Geirhos et al. [2021], which was 863 ms) from stimulus onset to final response. This suggests that our methodological choice of employing a touchscreen successfully reduced the RTs and thereby the time available for any form of (recurrent) higher-level processing.

**Figure 8.**
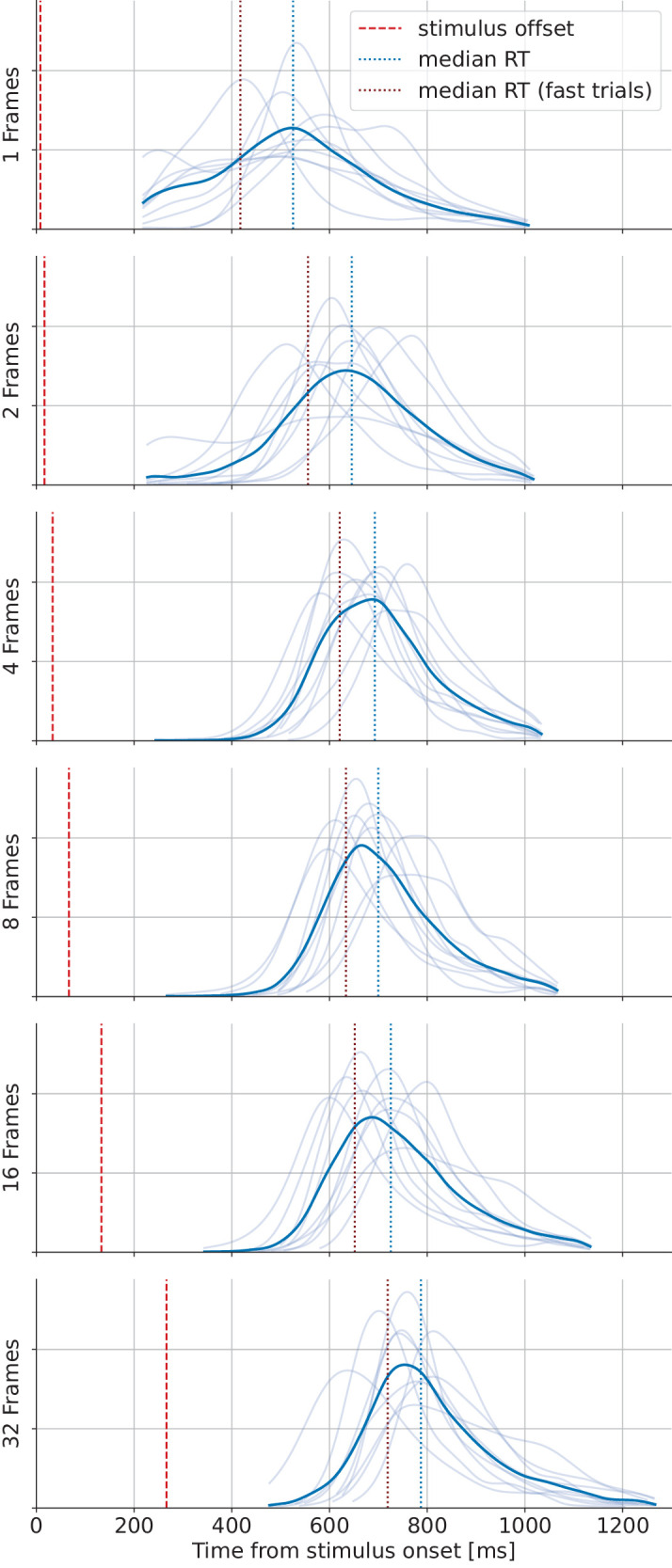
**Reaction times in milliseconds after stimulus onset.** We plot kernel density estimates of the reaction time for individual observers (faint dotted lines) and averaged across them (thick solid line). We also plot the median RT in each condition, as well as the median RT of the faster half of the median-split data.

It is possible that observers would have been able to give even faster responses if not for the 200-ms mask. For comparison, Thorpe et al. (1996) and Wichmann, Drewes, Rosas, and Gegenfurtner (2010) measured about 450 ms for an easier binary task and the faster response modality of lifting a finger off a button. Please note, however, that our task was an eightfold classification task, which, according to Hick's law (Hick, 1952, which relates the response time in *n*-alternative forced choice (nAFC) tasks logarithmically to the number of choices), requires longer response times purely on decisional grounds, after sensory processing. We are thus reasonably confident that in our experimental setting with the backward mask and the short response times obtained, the amount of top-down processing was limited. In Figure 8, we plot kernel density estimates of reaction times for the different stimulus presentation times for uncorrupted images. Evidently, the reaction times for longer presentation times are shorter, apart from the shortest condition of only one frame, presumably because observers resorted to guessing and just pressed a random symbol as soon as possible. For the longer presentation times, observers could initiate their response while the stimulus was still being shown, yielding shorter response times (and, as a corollary, limited any top-down processing).

**Figure 9.**
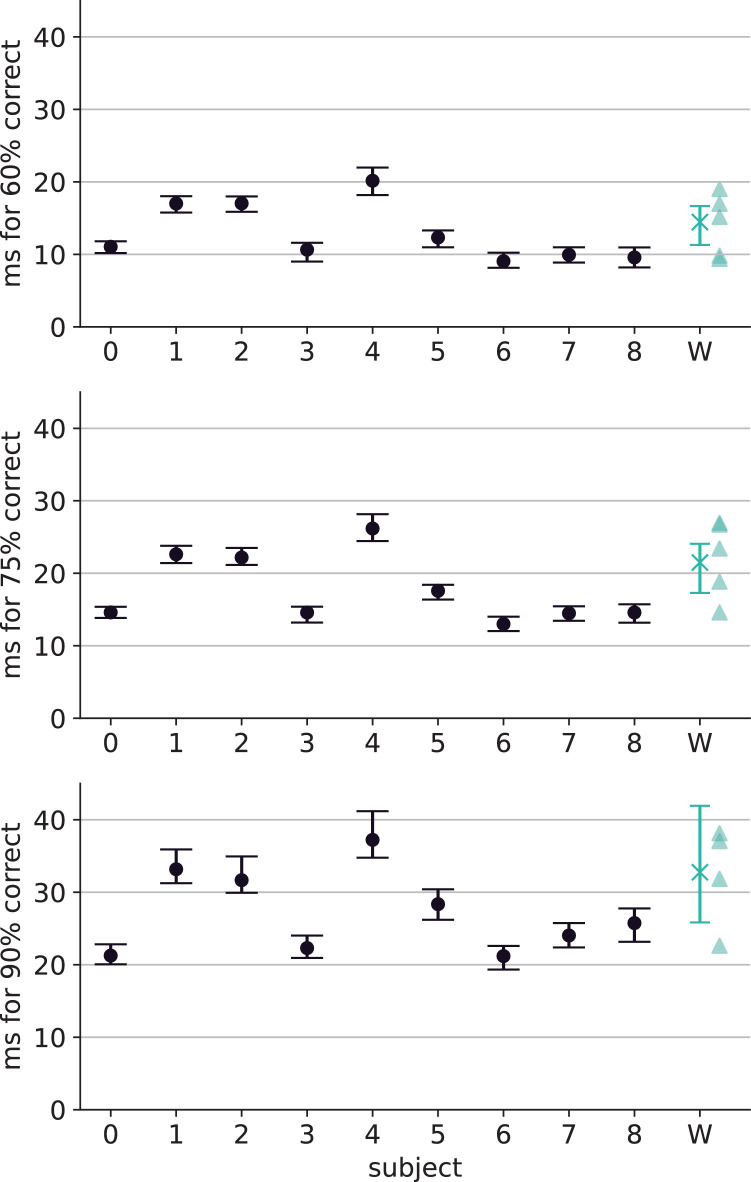
**Presentation times required to achieve 60%, 75%, or 90% proportion correct.** Analogous to Figure 5 in Wichmann et al. (2010), we plot the stimulus presentation times our observers needed to achieve certain performance thresholds in our (artificial) animal detection experiment with 68% confidence intervals. In addition to our nine observers (black dots), we plot the individual thresholds (turquoise triangles) and mean threshold (turquoise x) of their five observers, labeled as “W.” We fit a reverse Gumbel distribution and obtain a MAP estimate using the Python implementation of the Psignifit toolbox. Here, we used all data from the first iteration of the experiment, in which observers only had 800 ms to give their responses.

To contextualize our results, we render our results comparable to classic rapid animal detection tasks by collapsing our four animal categories (bird, dog, bear, elephant) and our nonanimal categories (bicycle, car, boat, airplane) to crudely obtain an animal detection task. We can then fit psychometric functions of binary classification performance and compare our findings to those of Wichmann et al. (2010), who report the stimulus presentation times required to achieve certain proportions correct (60%, 75%, or 90%). As can be seen from Figure 9, our results match the values expected from literature very well: We find object recognition performance with stimulus presentation times below 20 ms for 60% correct and around 30 ms for 90% correct—not only rapid animal detection but also rapid object recognition in general.

In summary, we demonstrate that the rapid recognition capability of the human visual system is not limited to simple binary tasks like animal detection, for which claims have been made that simple image statistics might provide sufficient discriminative information (Torralba & Oliva, 2003; but see Wichmann et al., 2010, for a counterargument). Instead, humans can rapidly solve even complex multiclass identification tasks with extremely short presentation times (≤17 ms for performance clearly above chance) and backward-masked stimuli with high accuracy.

### Observers are not more consistent at shorter presentation times

**Figure 10.**
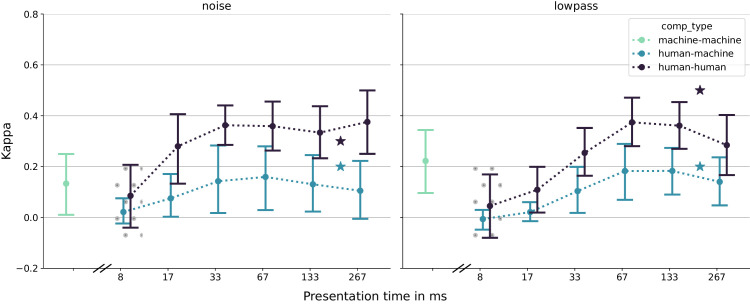
**Error consistencies as a function of presentation time for different corruption types.** Error bars indicate 68% percentile intervals of the bootstrapped distribution of EC values. Contrary to the initial hypothesis, human–human EC is not higher at shorter presentation times but instead quickly levels off at the values reported by earlier work. In the dotted region, the number of accuracy-maintaining flips is smaller than 10, rendering *κ* unreliable. The BagNet is not included in this set of models.

Next, we analyze error consistency (a) between different human observers, (b) between different DNNs, and (c) between humans and DNNs, as shown in Figure 10. Note that for the DNNs, images are simply fed forward through each network once (i.e., there is no notion of presentation time). We then compare the static DNN outputs to human responses for each presentation time.

First, we note that error consistency is a noisy measure: For each group of observers (e.g., DNNs, or several humans at some presentation time), the pairwise error consistencies span a wide value range. This is an inherent property of error consistency, which always requires a large number of trials to reliably converge to a stable value, highlighting the need to collect enough data to arrive at reliable estimates of the mean (Klein et al., 2025). This effect intensifies as the marginal distributions diverge from uniformity: When both observers perform near ceiling (as happens, e.g., for our human observers at long presentation times), *p_e_* approaches 1.0, so the numerator of Equation 1 is multiplied by a large factor (e.g., 20 if *p_e_* = 0.95). Thus, even a single changed response can have a large impact on the final *κ*, rendering the metric unreliable. In Figure 10, we plot error consistencies as functions of presentation time. We shade conditions in which EC is unstable by calculating the number of trials that would have to be flipped in one observer (from true to false or vice versa, while maintaining accuracy, i.e., always flipping a pair of responses) to achieve *κ_max_*. Low values mean that a few unlucky trials could have led to drastically different results. We shade the region where the number of flips is smaller than the somewhat arbitrary but conservative threshold of 10. Evidently, our experiment was robust to this effect, as the instability only affects the 8.3-ms condition. For uncorrupted images, the preconditions for the reliable calculation of error consistency are not fulfilled, proving that our corruptions are indeed necessary—see Supplementary Appendix C for a figure and further explanation.

We therefore turn to noisy and lowpass-filtered images, where we can calculate EC more confidently. At the shortest presentation time of only 8.3 ms, where humans perform at chance level because the stimulus is barely visible, EC takes on values of about 0, as expected. At the longest presentation time, our values roughly match earlier work, again in line with expectations (see Figure 1). But in between, and contrary to the “reconciliation hypothesis” that EC may be higher at shorter presentation times, we find that EC increases with presentation time and never exceeds the values reported by Geirhos et al. (2021).

These results imply that the discrepancy between great predictive power on neural data, on the one hand, and low error consistency to human observer behavior, on the other, is unlikely to be explained by observer-internal noise or feedback processes. While we cannot fully rule out that some recurrent processing takes place during our experiments, we take great care to limit the influences of such processes as much as possible: We not only present images for a very short amount of time but also force observers to give their responses within a very short window (800 ms) and interfere with recurrent processing by backward-masking the images with a high-contrast mask. These experimental design choices allow us to record behavioral responses with a median RT of 694 ms from stimulus onset to final response. We conducted a small experiment measuring the raw motor response time, consisting of the eight response options as they were shown in the main experiment, with a random one highlighted as a target that observers needed to press as quickly as possible. This experiment yielded a median RT of 367 ms, upper-bounding the time available for (possibly recurrent) processing to about 300 ms. We can further tighten this bound by also factoring in the time a signal takes to arrive at inferior temporal gyrus (IT): Image-selective neuronal activity is not measured earlier than 100 ms after stimulus onset (Thorpe et al., 1996; Johnson & Olshausen, 2003; DiCarlo et al., 2012), implying that these recurrent, observer-internal processes would have to be completed within about 200 ms. In any case, we *cannot* simply increase the measured consistency by showing images for a shorter amount of time to reduce processing hypothesized to cause diverging behavior—whether the divergence is believed to be, for example, through high-level knowledge and biases, feedback, or recurrent processing.

This analysis provides two insights: First, it further supports the results by Geirhos et al. (2018) by demonstrating that their experimental paradigm is robust with respect to the parameter of image presentation time, since qualitatively, their result that humans are more consistent with each other than they are with DNNs already holds after presentation times of only 17 ms. Second, it shows that the “reconciliation hypothesis” of DNNs as good models of the ventral stream only up to a point, after which recurrent processing of the extracted representations drives individual differences, is not supported by our data: We never observe higher error consistencies between human observers and DNNs at shorter presentation times.

### Removing ambiguous images does not qualitatively change results

**Figure 11.**
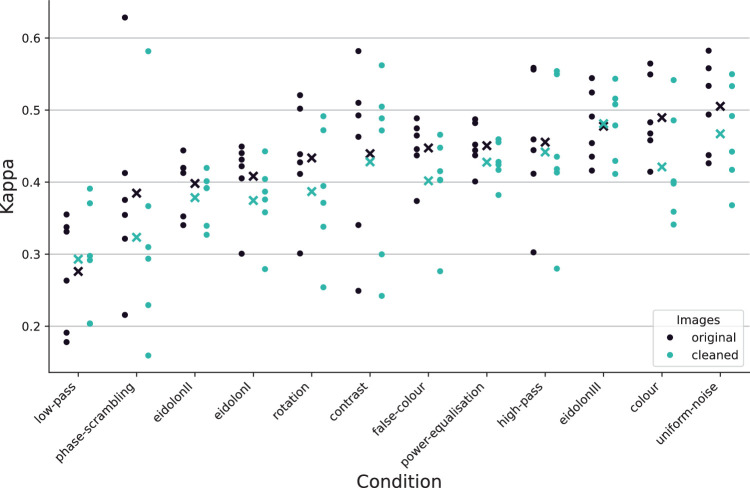
**ECs reported by earlier studies before and after the removal of ambiguous images.** Every scattered point corresponds to one pair of human observers; x-markers indicate the group mean. For every condition, we only visualize the data for the lowest corruption level, corresponding to no corruption (i.e., grouping by condition is only done to enable comparisons to Figure 9 from Geirhos et al., 2021).

To gauge the impact of removing ambiguous or otherwise problematic images on error consistency (as described in Supplementary Appendix A), we reanalyzed the original data from Geirhos et al. (2021) by manually applying our image cleaning paradigm to the images used in their experiments and recalculating error consistencies for only those images that would have passed our quality assurance. As Figure 11 shows, the impact of our cleaning procedure is overshadowed by the inherent noise level of behaviorally measured error consistency. The average EC is 0.43 ± 0.09 for original images and 0.40 ± 0.09 for the cleaned ones. The results by Geirhos et al. (2021) do not significantly change even if ambiguous images are removed, suggesting that the differences in EC on uncorrupted images between our work and theirs cannot only be attributed to ambiguous images. Probably, they instead stem from the fact that their 16-way classification task is much harder than our 8-way task.

### Model design choices hardly affect EC

Next, we investigate whether different model design choices (e.g., size, architecture, or training paradigm) lead to differences in error consistency—for example, one might hope that CLIP-trained models, which receive rich textual labels and are trained on large datasets, are more human-like than models that were simply trained in a supervised fashion on smaller datasets like ImageNet and with sparser labels such as class-level annotations.

**Figure 12.**
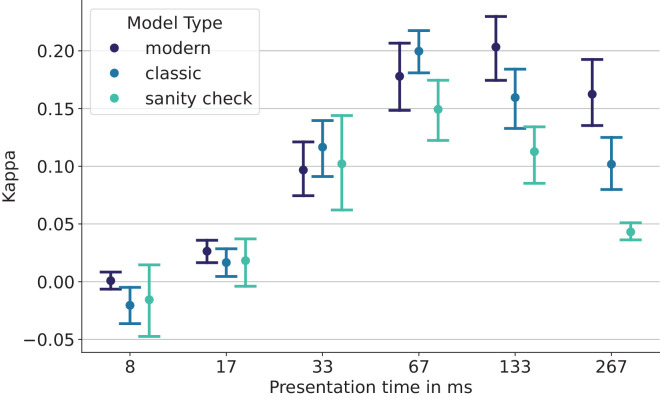
**ECs on lowpass-filtered images by model.** We plot the average EC to humans for all three model types. The BagNet, which we used as a canary, is indeed the least human-like model. As presentation time increases, the gap between modern and classic models widens.

In Figure 12, we break down human–machine error consistencies by model on lowpass-filtered images (see Supplementary Figure S9 for plots on uncorrupted and noisy images). For every presentation time, we scatter every model's EC to human observers. However, even ignoring the intrinsic uncertainty of the EC values, no clear trends emerge as to which models are best. What can be said with some confidence is that AlexNet, BagNet, and EfficientNet are the worst models, simply because of their lack of robustness. But beyond the similarity gained through better performance, there does not seem to be a reliable relationship between how a model was trained, or on what data, or how large it is, and its error consistency to human observers. This result reflects the Brain-Score benchmark, where very different models also achieve similar scores.

### Images should be drawn from the validation set

**Figure 13.**
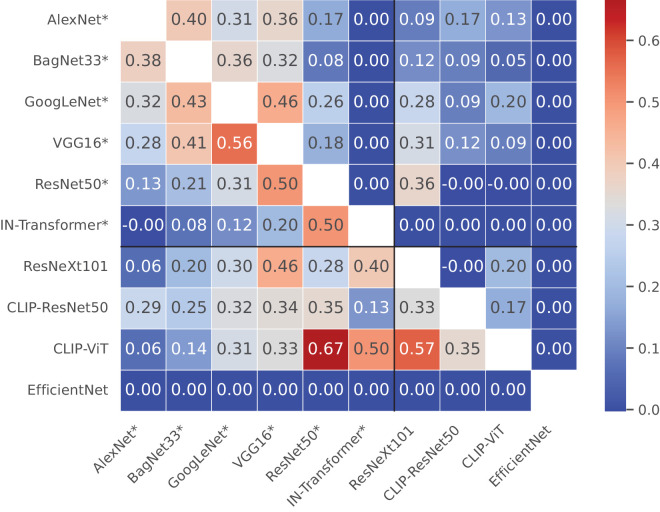
**Matrix of pairwise error consistencies between models. Each cell contains the EC for a pair of models.** In the upper half of the matrix, we show the EC over all training set images. In the lower half of the matrix, we instead use all validation set images. Models trained on ImageNet are denoted by asterisks. Contrary to the expectation that EC might be higher for the training set, we actually find higher values on average in the lower half, for validation set images.

In earlier experiments (Geirhos et al., 2018; Geirhos et al., 2021), all images used to construct stimuli were taken from the training set of ImageNet-1k, instead of its validation set. Arguably, since these images are independent and identically distributed, this should not matter for human observers. But for models trained on ImageNet-1k, this decision could have an impact on model behavior: Because the ground-truth label is used during training, a model could overfit to certain training set images and learn to respond correctly to these images specifically, instead of learning a robust decision boundary that generalizes to unseen images. Consequently, error patterns on the validation set could differ from those on the training set. The larger the train–test gap, the larger the potential for such differences. We empirically test the impact of the stimulus source by evaluating the error consistency between all DNNs in our suite of models and find that the choice of image set does indeed drastically affect error consistency in the absence of corruptions. Error consistency values on validation set images are, on average, *larger* than on training set images, not smaller, as one might have expected (0.33 vs. 0.19, after dropping DNNs that would skew results by not making any mistakes). Furthermore, there are pairs of models with completely different error consistency values for the different image sets (see Figure 13). For example, the EC between the ResNet-50 and the CLIP-ViT is 0.67 for validation set images but 0.0 for training set images, even though both image sets are very large (>1,000 images) and values for each set should be relatively stable. This finding illustrates that EC is not an innate property of a pair of observers but is defined relative to a dataset. For the noisy and lowpass-filtered images, there are no such differences in the mean consistencies of training and validation set images, and the train–EC/validation–EC pairs are highly correlated (Pearson's *r* > 0.93, *p* < 1 × 10^−12^). In other words, for sufficiently corrupted images, *κ* as measured on training set images is an almost perfect predictor of *κ* on validation set images, so it does not matter which one is used. But for uncorrupted images, the correlation is much weaker (Pearson's *r* = 0.256, *p* = 0.188), so there does seem to be a systematic influence of image source. This matches our intuitions, since for sufficiently strong image corruptions, it no longer matters whether the base image was drawn from the training set, as the corruption changes the image so much that it effectively becomes out of distribution. See Supplementary Appendix D for more detailed results. In summary, we find that the choice of the stimulus source can cause issues with error consistency, not only by changing observer accuracy to the point of numerical collapse but also by introducing artifacts that are removed by applying image corruptions. Further work should use validation set images to assess error consistency or corrupt images sufficiently to render them dissimilar enough from the training set, as was done here.

## Discussion

We present an analysis of the influence of stimulus presentation duration on error consistency between humans and deep neural networks. We find that (a) results of earlier studies (Geirhos et al., 2018; Geirhos et al., 2021) are fairly robust to the choice of presentation time and that (b) error consistency is not higher at shorter presentation times, suggesting that differences in classification decisions between humans and machines are unlikely to be explained by the influence of observer-internal idiosyncrasies, for example, through feedback-mediated memory. In fact, human observers already agree with each other about the difficulty of uncorrupted natural images after only 17 ms of exposure to the stimuli. Even if we focus our analyses on the faster half of median-split data, resulting in response times that are 80 ms faster on average, our error consistency results do not meaningfully change, leaving little time for higher-level mental processing.

All 10 investigated DNNs show the very consistent trend of being less behaviorally similar to human observers than human observers are to each other, across all presentation times. If these DNNs were good models of the early visual system, and the behavioral differences between humans and DNNs were only caused by top-down processing of the extracted representations, we might expect shorter processing times to lead to more similar behavior. But this is not the case, and since the vision models we investigated here cover such a large portion of the domain of DNN design choices, we are fairly confident that our findings generalize well (i.e., that there is no other performance-optimized model that reliably makes more human-like errors).

Further work could evaluate human–machine consistency not only on images with a clearly defined ground-truth label but also on synthetic images for which no such labels exist (e.g., Rohrschach-like images). Presumably, the differences found between humans and machines in such experiments would be way larger than the ones we report here, because models were trained to classify natural images correctly. However, this would also necessitate new methods of quantifying consistency, because calculating error consistency requires ground-truth labels.

In conducting this analysis, we were able to establish that human observers are not only capable of solving binary recognition tasks “ultra rapidly” with high accuracy but can also solve an eightfold identification task “ultra rapidly” with high accuracy, even in the presence of image corruptions and restricted viewing time, as well as backward masking. This finding adds to the growing body of work characterizing the human visual system as a fast and robust feed-forward feature extractor.

Still, a methodological limitation of this work is that while we try to suppress recurrent processing in human observers as much as possible by backward-masking the stimuli and providing only very short response windows, we ultimately still record behavioral responses. While we shortened the time window available for recurrent processing to a maximum of 200 ms, we cannot ascertain that no recurrent processing of the extracted representations takes place, possibly in parallel to the motor-cortical preparation of the response itself. To overcome this limitation, one would have to employ other experimental methods with high temporal resolution, such as EEG (Thorpe et al., 1996) or saccadic choice tasks (Crouzet, Kirchner, & Thorpe, 2010), to extract classification decisions early enough to rule out any recurrent processing.

## Supplementary Material

Supplement 1

## Footnotes

1
https://vpixx.com/datasheets/ds_touchpixx.pdf.

2 We also ran a small experiment with a colored pink noise mask, finding that this mask is not more effective at masking the stimuli (see Supplementary Appendix A).
