# Supplementary material for: Behavioral differences between humans and machines arise early in visual processing
Source: J Vis. 2026 Feb 17;26(2):9. doi: 10.1167/jov.26.2.9 (PMC12922715; doi:10.1167/jov.26.2.9)
Supplement: Supplement 1 [file jovi-26-2-9_s001.docx]

# Supplementary Materials

## Stimuli

The original stimuli used in earlier experiments suffer from data quality issues, some of which are exemplified below. There are also entire categories of images that might be considered problematic. For example, the category *oven* contains many images of rotisserie chicken, which might confuse a naive observer or even prompt the wrong label *bird*, and the category *cat* contains big cats like lions, which might semantically be closer to the category *bear* than they are to house cats. The other issues can be grouped into four stereotypical problems:

1. *ambiguous labels* captures images containing objects belonging to more than one of the available category options.
2. *problematic cropping* captures images for which the 224 × 224 center crop presented to observers crops out the object that drives the true label.
3. *label errors* captures images that do not fit any of the categories properly, due to label errors in ImageNet.
4. *confusing scene* is a catch-all category for other, vaguely problematic images where even an observer without a time constraint would be unsure what the correct label is supposed to be.


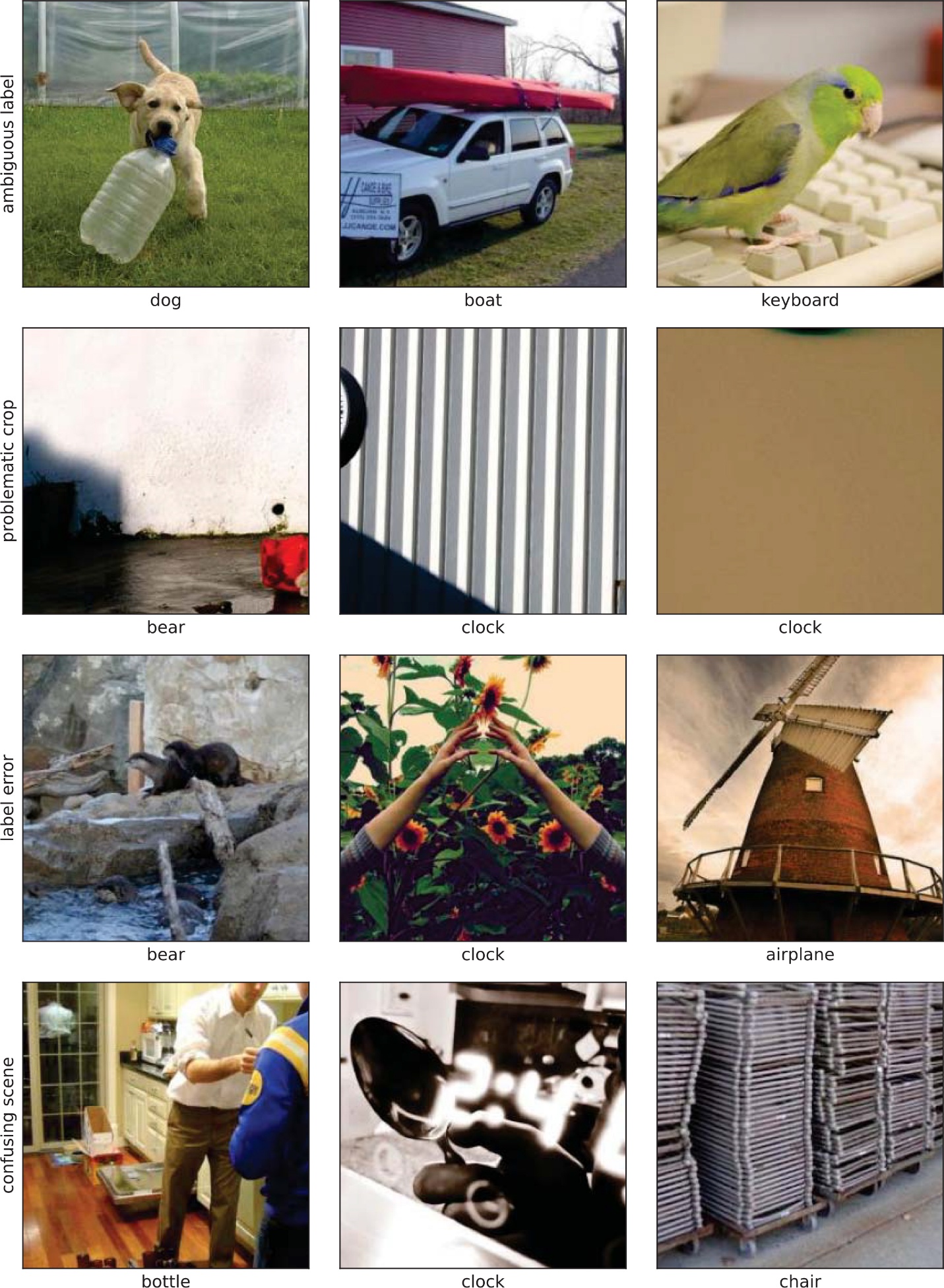


**Supplementary Figure S1**. **Prototypical examples for problems with the original stimuli.** The annotation indicates the ground-truth label of the image. **First row.** Dog, bottle, car, boat, bird and keyboard are all possible response options in the original experiment, but for these images, it is not clear what the correct response should be because both objects are present. **Second row.** All images are shown as a 224 × 224 pixel center crop, because this is how images will be presented to the models as well. But sometimes, cropping will cut out the object of interest, resulting in non-informative images. **Third row.** For some images, the ground-truth label is simply wrong: Neither of these images belong to any of the possible categories. **Fourth row.** Many images do technically contain the ground-truth label, but the scenery is so confusing and unclear that even an observer without any time constraints might struggle to correctly classify such images.

The original 16 categories were therefore reduced to the following eight unproblematic categories: airplane, boat, car, bicycle, elephant, bear, dog, bird. This reduction of categories makes the task easier for humans, and the buttons used for indicating classification choices can all be placed in an area of the screen that is easy to reach. It also removes categories that are problematic, such as oven and cat. To be included in the experiment, a candidate image needs to fulfill the following requirements:

1. The object is present and not fully cropped out, i.e. the label is correct. Partial crops are okay, as long as the object can still be recognized (for cyclists, this means that some part of the bicycle needs to be in the image—two cyclists in gear and helmets, but without the bike below them, is not sufficient for inclusion).
2. No instance of any of the other 15 categories is present, e.g., if a dog image contains a cat, we exclude it, even though we are not planning on keeping cats. This decision was made to give us flexibility, if for some reason we decided to use other categories later.
3. The image is not ambiguous, i.e. an observer with unlimited time is certain about the category label.
4. The image is not profane, i.e. contains no nudity / gore / etc.
5. The image is not a collage of multiple images (as this would interfere with the center fixation location).
6. No internal perspectives: for both airplanes and cars, images taken from within are so rare (and unlike the other images of the category) that we exclude them.
7. No abstractions: a very realistic toy model of a car is okay, but a stone carving of an elephant or bird-origami are not, for example.
8. No non-prototypical examples: A bicycle has, by definition, two tyres. Tandems are okay, but custom one-of-a-kind 4-wheeled contraptions are not.

**
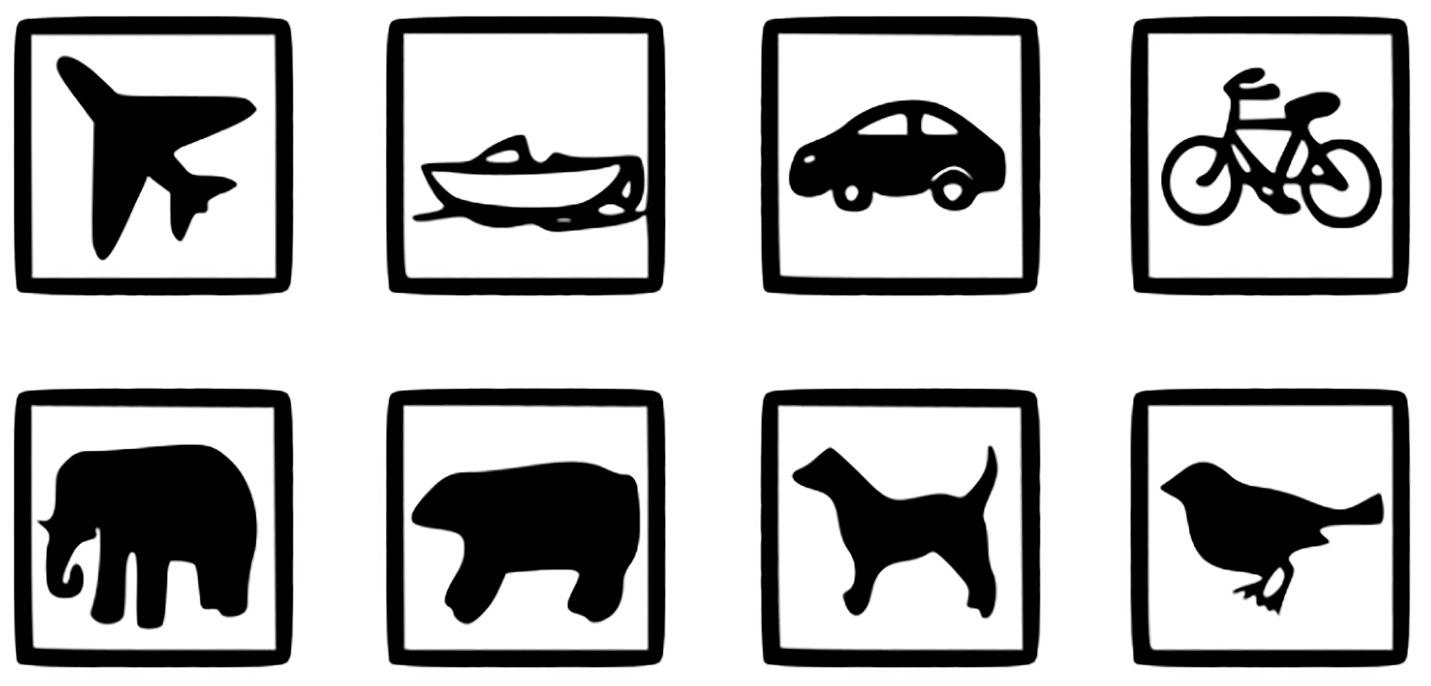
**

**Supplementary Figure S2**. **The response options presented to observers.** This arrangement of icons was displayed on the touchscreen as soon as the presentation time of the pink noise mask was over.

**
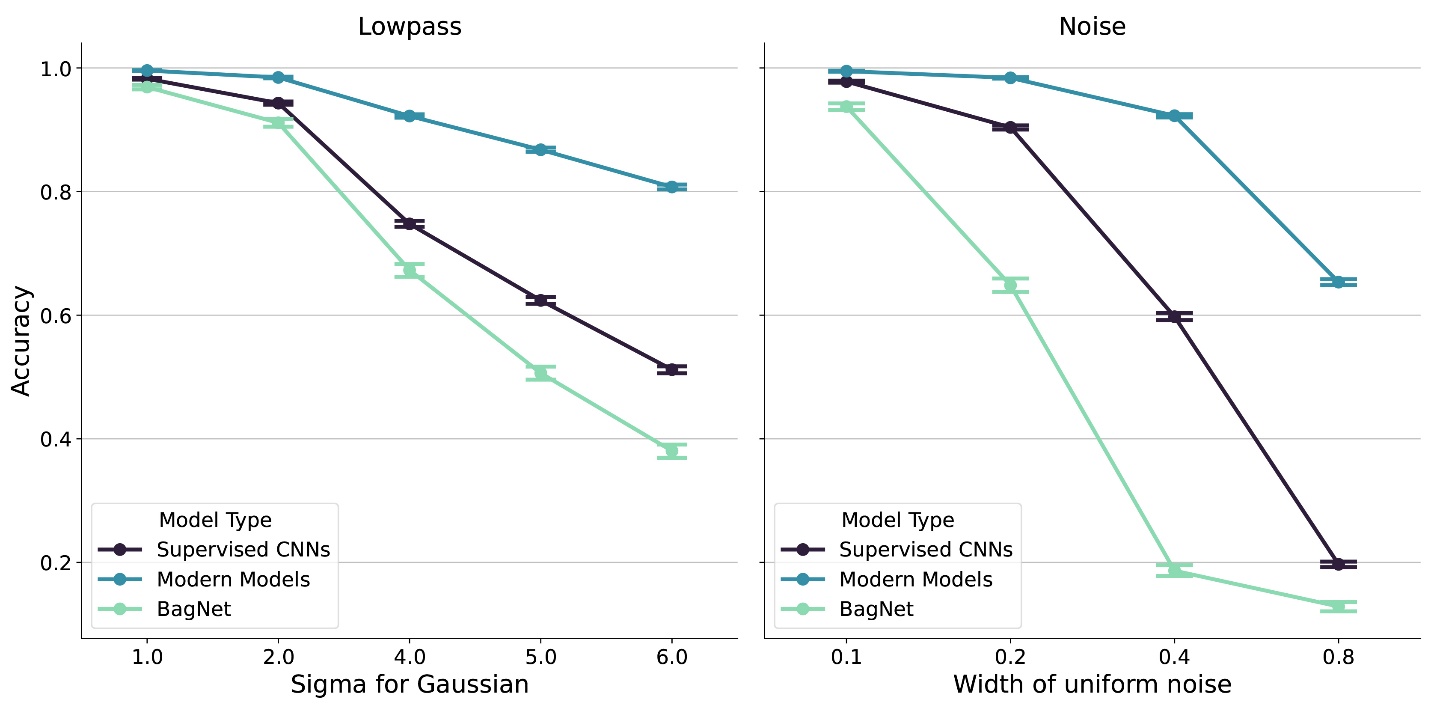
**

**Supplementary Figure S3**. **The effects of corruption parameters on model performance.** We plot the average accuracy achieved by the three groups of DNNs (supervised CNNs and modern models, with BagNets as sanity check) against the parameters of the corruptions. **Left.** The σ of the Gaussian filter used to blur images. **Right.** The width of the uniform noise to be added to all channels.

**
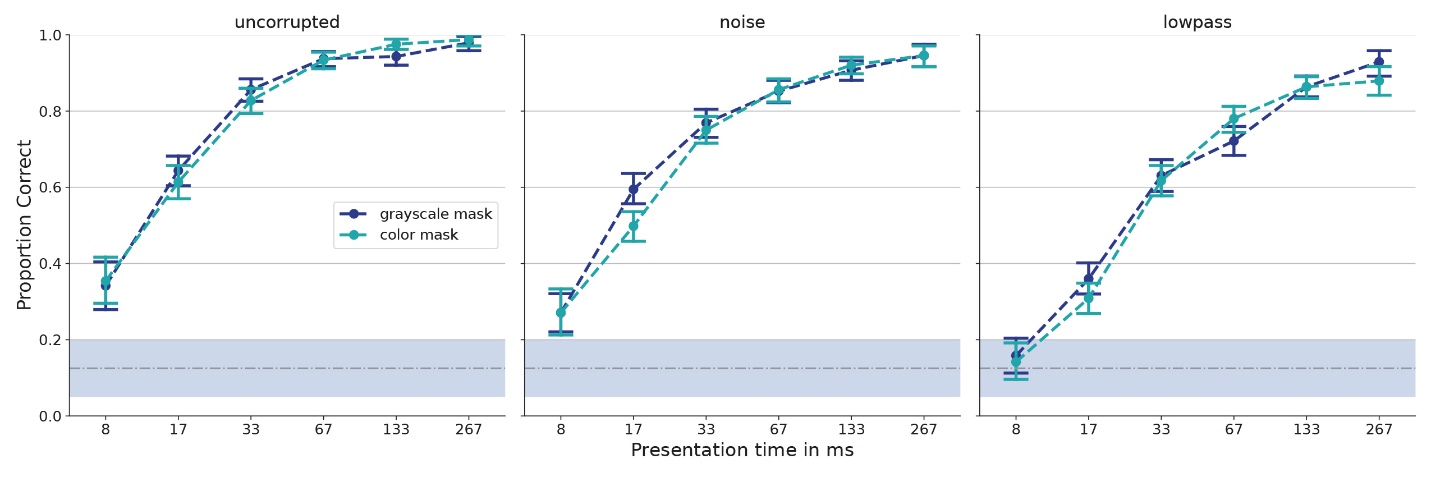
**

**Supplementary Figure S4**. **Effects of using a color mask.** To check whether the grayscale pink noise mask employed in the main experiment was sufficient for masking our colored stimuli, or whether a color mask would have been necessary, we conducted an additional experiment in which the first author and two students from the lab (both experienced observers) were tested with different masks. We tested a color mask obtained by stacking three pink noise masks like the ones used in the main experiment, resulting in a color mask with lower RMS luminance contrast than the main mask. To remove this confound, we then obtained the empirical distribution of RMS contrast of luminance, assuming the channel weights of the Rec. 709 standard. Using this data, we created a matching grayscale pink noise mask by scaling down the contrast. Evidently, while the performance of these experienced observers at lower absolute mask contrast was better than that of participants in the main experiment, there is hardly a substantial difference between the conditions, convincing us that the grayscale mask was indeed sufficient.

## Response Times

Initially, we conducted the entire experiment with a maximum response time of 800 ms for every trial, because pilot experiments suggested that this would be sufficient. Keeping the response times low enabled us to collect a complete set of responses in 4 hours per observer, and further restricts observers’ processing time. However, the human-human EC at longer presentation times (133 and 267 ms) dropped below the levels reported in earlier work, where a 1500 ms window was offered to give a response. We then tested three observers at 17, 67 and 267 ms with a 1500 ms response window. This experiment revealed that at the longer presentation times, when almost no real mistakes are made, the few random mistakes that are caused by motor noise or the touchscreen not registering responses properly drastically decrease the measured EC. We therefore re-collected responses at 133 and 267 ms with a 1500 ms response window, allowing observers to tap the touchscreen again when it failed to register the first response, which mostly alleviates the problem. At shorter presentation times, the touchscreen presumably fails with a similar frequency, but enough actual mistakes are made to render EC robust against these trials.

## Idiosyncrasies of Error Consistency

As discussed in Section 2, the metric we use to measure behavioral similarity is—though fairly well established in the context of human-machine comparisons (Geirhos et al. 2021, 2020)—characterized by a few idiosyncrasies practitioners should be aware of. For an in-depth treatment, see Klein et al. (2025). We compare different alternatives, as discussed below, in Supplementary Figure S6.

The main complication for works such as ours, where EC values for different pairs of observers are directly compared, arises from the fact that the bounds for EC (i.e. the minimum / maximum EC that a pair of observers can achieve) depend on the marginal correctness probabilities of the observers. Two observers with very different accuracy levels can only reach *κ* ≤ *κ_max_*, with *κ_max_* < 1.0. For example, a state-of-the-art DNN with 95% accuracy and a human at short presentation time with 60% accuracy cannot achieve *κ* > 0.15. As said before, it is not clear whether this property is a bug or a feature, since it were arguably somewhat absurd if a very inaccurate observer could be more error-consistent with an accurate one than another high-accuracy observer. For this work, we make sure that all preconditions for the successful application of error consistency are met by introducing corruptions (noise and lowpass filtering) to assimilate observer accuracy. We illustrate the importance of this step in Supplementary Figure S5, where we plot error consistencies on uncorrupted images but shade regions where (a) the empirically measured human-human EC exceeds the maximum attainable human-machine EC and (b) the empirical EC values should be considered unstable, because the number of accuracy-preserving response flips is lower than 10 (i.e. if we were to allow ourselves to rearrange classifier responses to increase their EC without changing their accuracies, exchanging fewer than 10 responses would be enough to attain *κ_max_*).

**
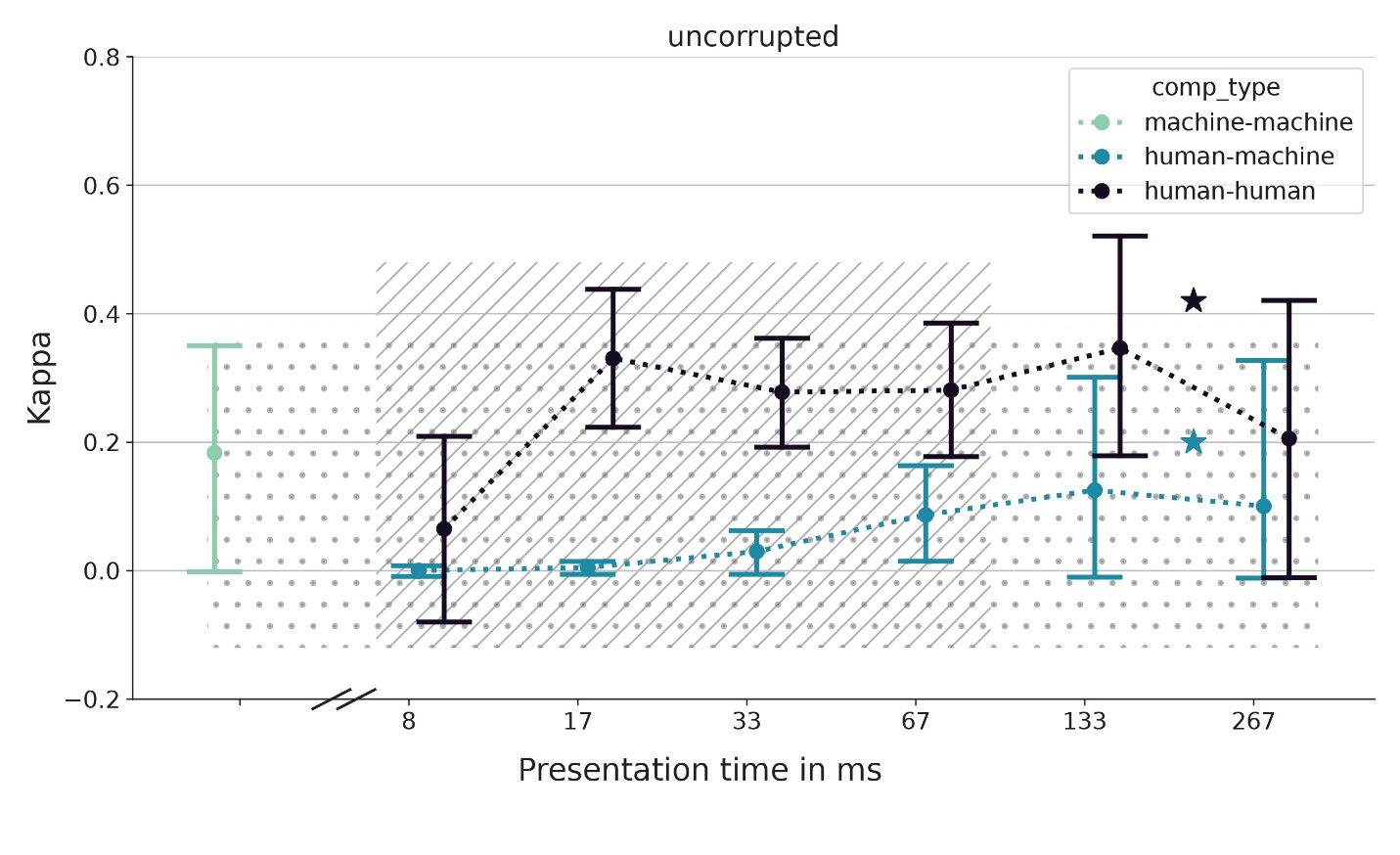
**

**Supplementary Figure S5**. **Importance of corruptions.** On uncorrupted images, the preconditions for reliably calculating EC values are not met: In the dotted region, there is at least one comparison where EC is unreliable because the number of accuracy-preserving flips is smaller than 10. In the hatched region, the empirically measured human-human EC exceeds the maximum theoretically attainable human-machine EC. By applying corruptions to our images in the main experiment, we avoid these issues almost completely.

One could try to address this issue in various ways, for example by naively dividing *κ* by *κ_max_* to normalize. For our data, this drastically increases the variance of human-machine consistencies on uncorrupted images, due to the aforementioned ceiling and floor performance issues, but agrees with EC on corrupted images. Other attempts have been made to formulate a metric that is by design always bounded by [−1, 1] irrespective of the marginals. For example, Safak (2020) propose Min-Mid-Max scaling. Instead of $\kappa = \frac{p_{obs} - p_{exp}}{1 - p_{exp}}$, they propose $\kappa = \frac{p_{obs} - p_{exp}}{p_{max} - p_{exp}}$, i.e. scaling by the minimum and maximum agreement possible under the marginals. This method again does not lead to different conclusions on noisy and lowpass-filtered images.

Another noteworthy property of error consistency is that it does not take into account the precise nature of mistakes. Two observers will appear consistent even if they make very different mistakes, as long as they make them on the same images. For example, if for all dog-images, observer A responds cat while observer B responds bicycle, these mistakes are made on the same images and increase the measured error consistency, even though presumably, the processes the observers use to arrive at their respective classification decisions are quite different. A possible remedy to this issue has been proposed by Goel et al. (2025): They propose *Chance Adjusted Probabilistic Agreement (CAPA)* which calculates *κ* over the responses themselves (i.e. before comparisons to ground truth) while calculating the expected agreement from independent observers responding conditionally on the ground truth label of every sample. This way, different mistakes actually decrease the consistency. Again, calculating this metric for our data does not yield qualitatively different results to those presented in Figure 10.

Of course, the quantification of behavioral similarity is not limited to variants of Cohen’s Kappa. Walther (2013) propose an alternative based on the mutual information between the confusion matrices of classification decisions, which could be a very flexible approach. However, since it does not directly compare decisions for individual samples, it does not achieve the same level of granularity as the other consistency measures.

**
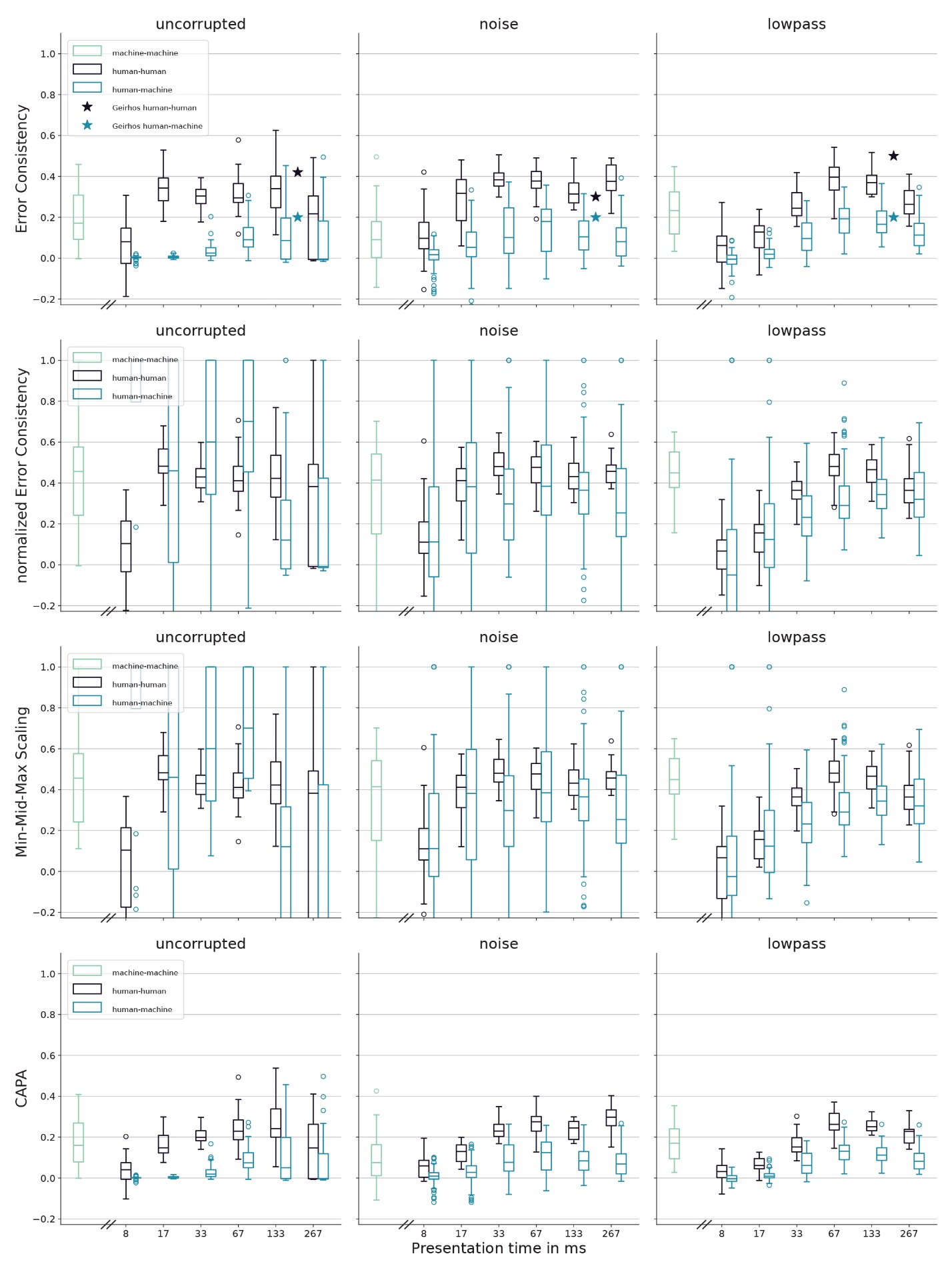
**

**Supplementary Figure S6**. **Different variants of quantifying consistency.** We investigate a series of different measures of observer consistency and find that they ultimately agree about the conclusion: DNNs and human observers are not more consistent with each other at shorter presentation times than they are at 250 milliseconds.

## Additional Results

**
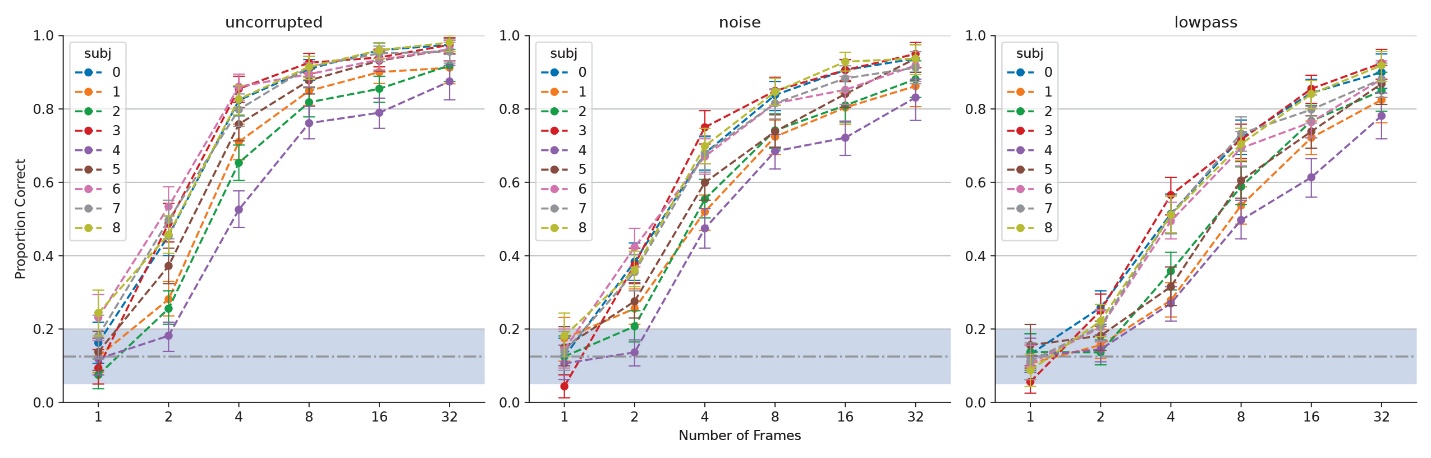
**

**Supplementary Figure S7**. **Performance comparison of all observers, broken down by image corruption.** This figure shows the same data as Figure 6, but color-codes observers to showcase that they perform consistently across conditions.

### Error Consistency for grayscale images

In Supplementary Figure S8, we present results for grayscale images, which closely match those we obtained for the other corruptions: Error consistencies increase, but never exceed the values reported in earlier work. Again, the values we measure at the longest presentation times are subject to instability, because at such high accuracy levels, EC can drastically change because of only very few trials. The same issue affects machine-machine consistency, because the models reach high accuracy on grayscale images.

**
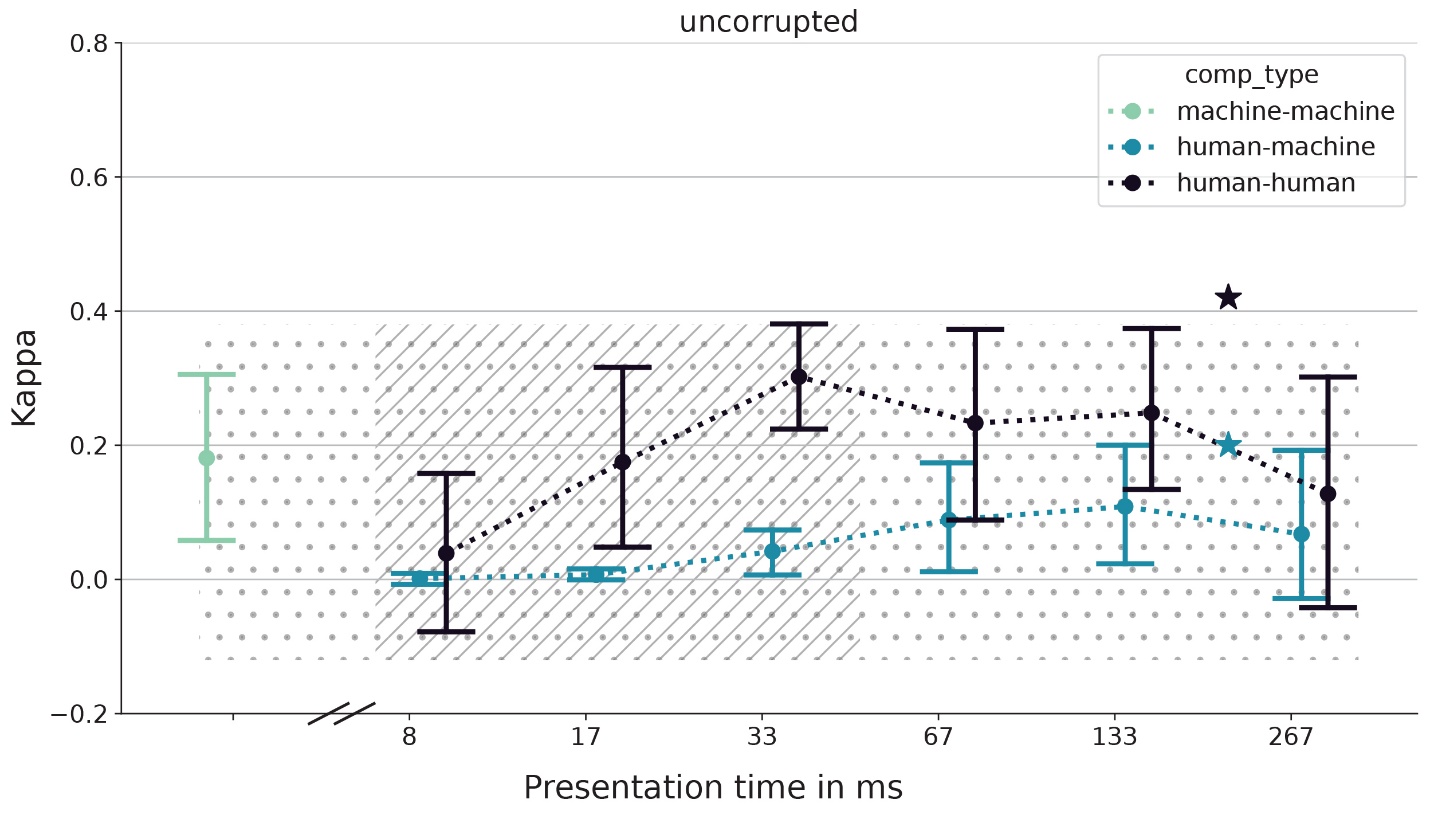
**

**Supplementary Figure S8**. **Error consistencies for grayscale images**. Figure analogous to Supplementary Figure S5: In the dotted region, there is at least one comparison where EC is unreliable because the number of accuracy-preserving flips is smaller than 10. In the hatched region, the empirically measured human-human EC exceeds the maximum theoretically attainable human-machine EC. Error Consistencies increase to, but never exceed the values known from literature. However, conditions for the reliable calculation of EC are not fulfilled.

### Error Consistency by Model

Next, we break down human-machine error consistencies by model in Supplementary Figure S9. On uncorrupted images, almost all models appear completely inconsistent with humans, because they make so few mistakes. But on the noisy images, differences between older and newer models emerge. Particularly BagNet—specifically chosen because we expected it to be quite inconsistent with humans—seems to achieve only very low consistency. This makes sense, because this model relies on local image structure for its classification decisions, which is disrupted by the noise. This sanity check shows that in principle, the metric does afford enough resolution to find differences between models. However, no such differences are found for the lowpass-filtered images.

**
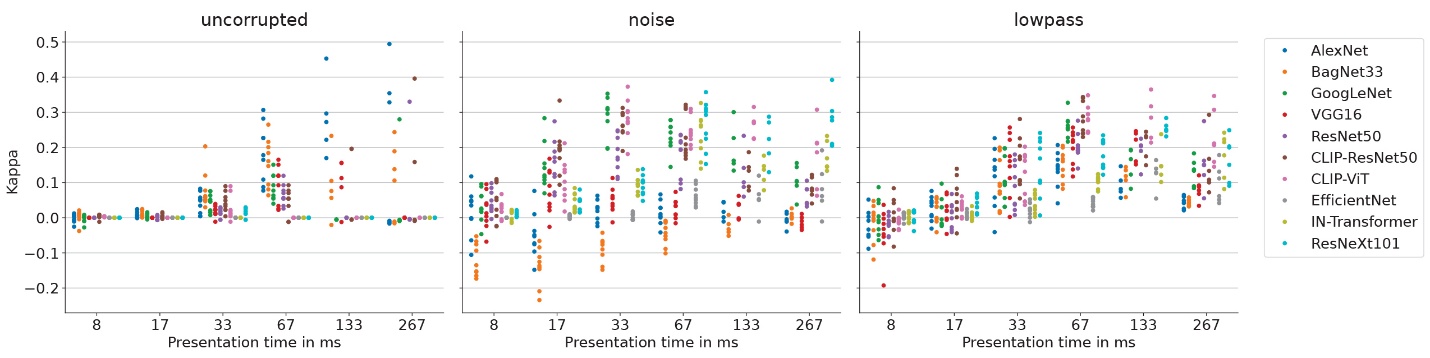
**

**Supplementary Figure S9**. **ECs by model.** Best viewed on screen, in color. Here, we take a closer look at human-machine error consistencies, broken down by image corruption type and model. Every point is a pair-wise error consistency between a human observer and a DNN.

### Effect of corruptions on output entropy

Since the investigated models were not trained on corrupted images, one might wonder whether the corruptions lead to atypical model responses that do not resemble the normal model classification behavior. Model decisions could collapse into always predicting the same class, or never predicting some classes at all. As a proxy for such issues, we consider the entropy of the models’ output distributions over the entire dataset in Supplementary Figure S10. For uncorrupted images, all models produce the desired uniform distribution over classes, leading to a maximum entropy of three bits. Evidently, introducing our corruptions only slightly decreases the entropy in most cases, and the only problematic collapses happen for the BagNet and two weaker models on noisy inputs. Since we also do not decay model performance to unreasonable levels (85% average accuracy), we are fairly optimistic that evaluating models on these distribution shifts is viable.

**
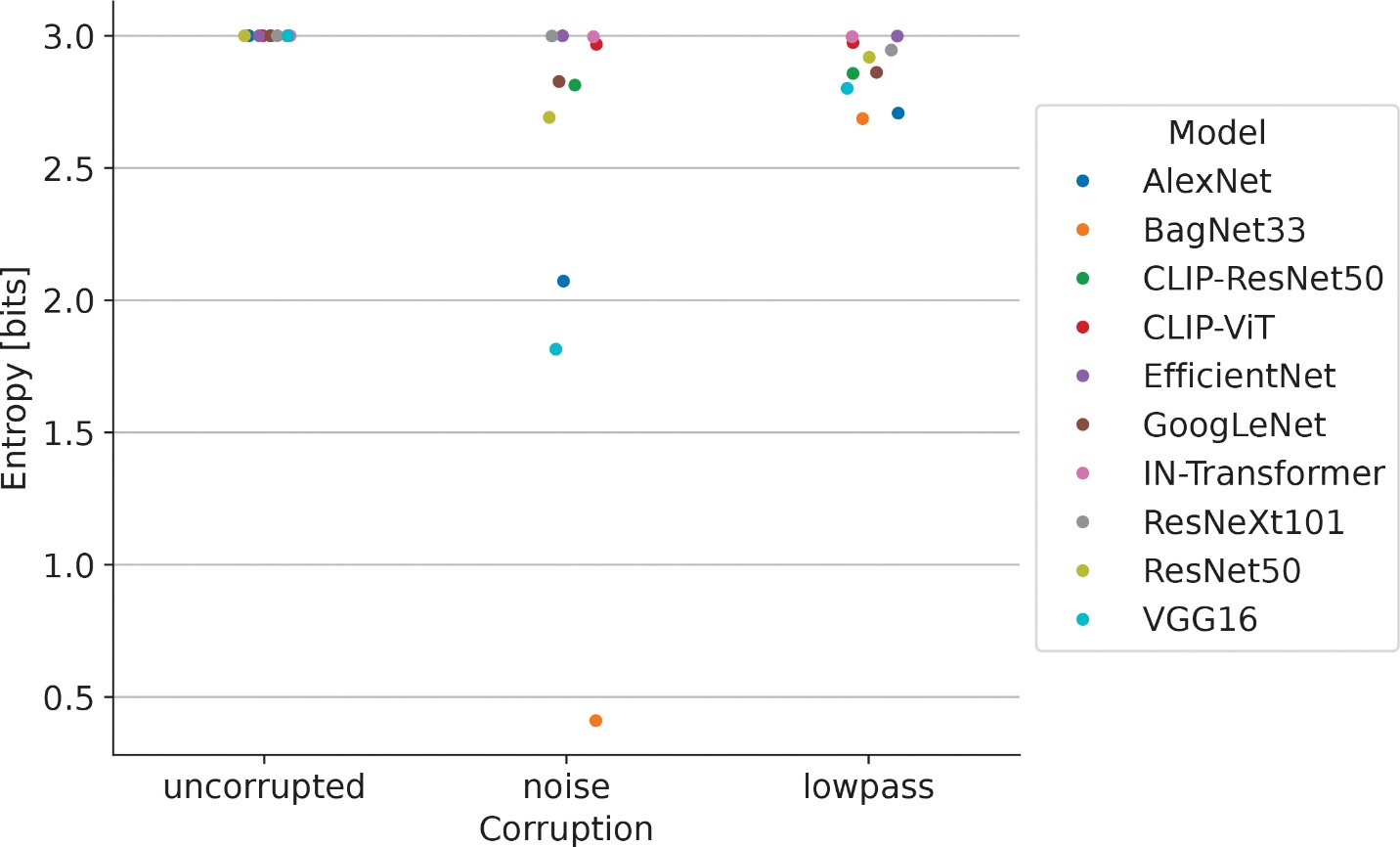
**

**Supplementary Figure S10**. **Model output entropy by corruption.** We plot for every corruption and model the entropy of the distribution of responses, in bits.

### Influence of validation set images

We have demonstrated that for uncorrupted images, it seems to make a difference whether images from the training set of ImageNet or the validation set are used. Here, we provide heatmaps analogous to Figure 13 for noisy and lowpass-filtered images. Furthermore, we scatter training set EC against validation set EC for all pairs of models in all three corruptions, demonstrating clearly that EC on training set images is not a good predictor of EC on validation set images *only for uncorrupted images*. This clearly hints at overfitting to some training-set images as a possible explanation for the difference.

**
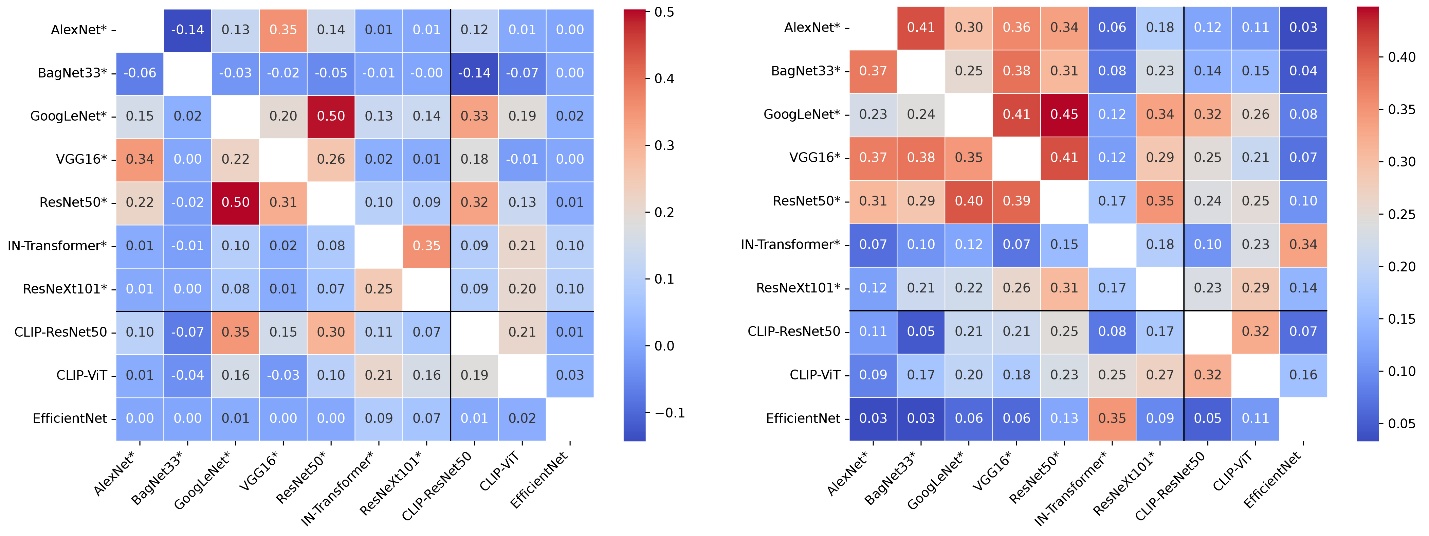
**

**Supplementary Figure S11**. Pairwise error consistencies on training- and validation set images, analogous to Figure 13. Each cell contains the EC for a pair of models. In the upper half of the matrix, we show the EC over all training set images. In the lower half of the matrix, we instead use all validation set images. Models that were trained on ImageNet are denoted by asterisks. **Left: noisy images.** **Right: lowpass-filtered images.** The differences between training set and validation set are far less pronounced than on uncorrupted images, see Supplementary Figure S12 for correlations.

**
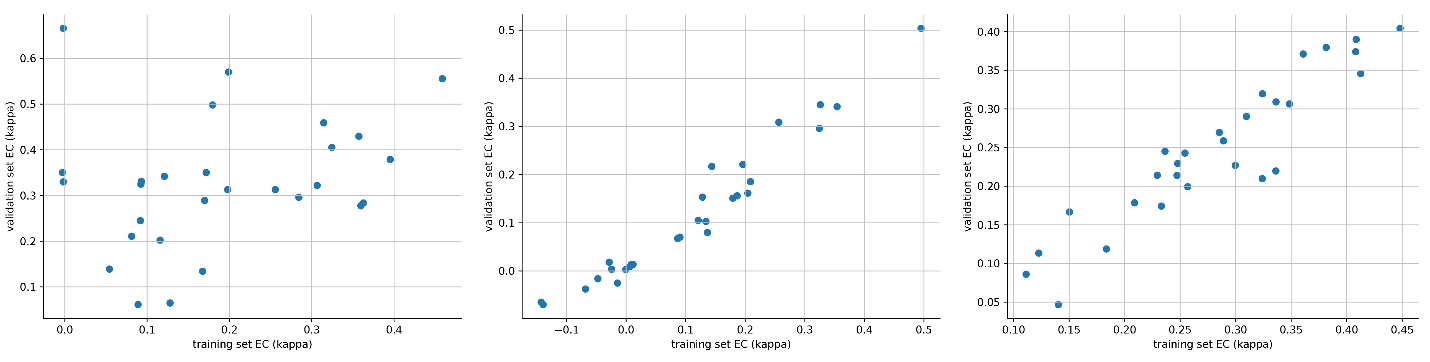
**

**Supplementary Figure S12**. Scatter plots of training set EC against validation set EC. **Left: uncorrupted images.** Evidently, error consistency on training set images is not a good predictor of error consistency on validation set images in the absence of corruptions (Pearson’s r = 0.256, p = 0.188). **Middle: noisy images.** The correlation between EC on the training and test set is very strong and highly significant (Pearson’s r = 0.973, p = 3.86  ×  10^−18^). **Right: lowpass-filtered images.** Again, we find strongly and significantly correlated error consistencies (Pearson’s r = 0.931, p = 6.42  ×  10^−13^).
